# Supplementary material for: Complete fusion of a transposon and herpesvirus created the Teratorn mobile element in medaka fish
Source: Nat Commun. 2017 Sep 15;8:551. doi: 10.1038/s41467-017-00527-2 (PMC5601938; doi:10.1038/s41467-017-00527-2)
Supplement: Supplementary file 1 — Supplementary Information [file 41467_2017_527_MOESM1_ESM.pdf]

Description of Supplementary Files

File Name: Supplementary Information
Description: Supplementary Figures and Supplementary Tables

File Name: Supplementary Data 1
Description: Fasta-formatted sequences of six individual *Teratorn* copies obtained by BAC sequencing.

File Name: Supplementary Data 2
Description: Sequences and gene annotations of *Teratorn* and *Teratorn*-like elements.

File Name: Peer Review File

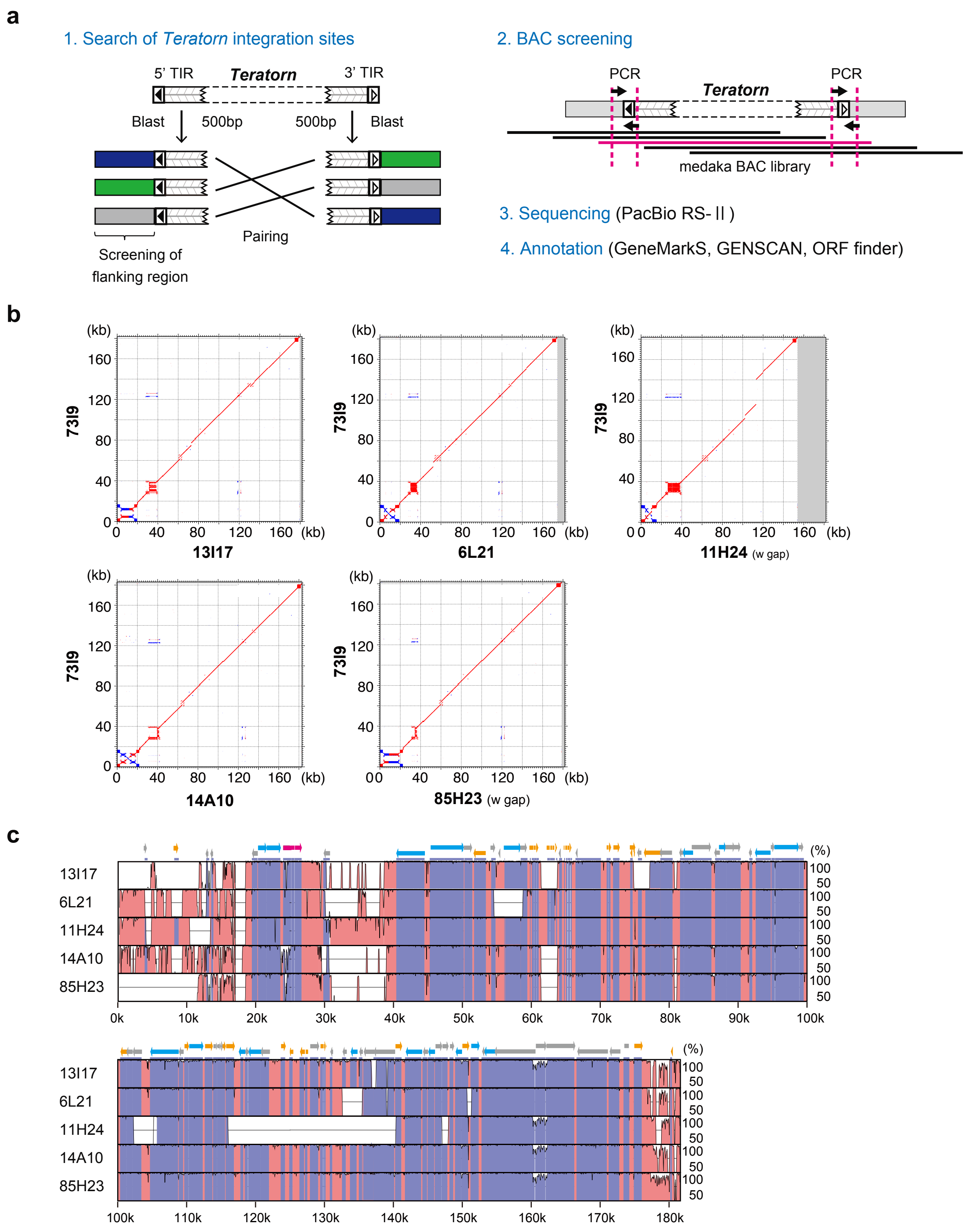

Supplementary Figure 1 | Global structure is almost the same among individual *Teratorn* copies, except for some indels. (a) The procedure of screening and sequencing of the full-length *Teratorn* copies: 1. *Teratorn* insertion site determination by screening of 5' and 3' flanking regions from the public medaka genome database, followed by pairing of the 5' and 3' flanking regions, which are derived from the same loci; 2. Screening of BAC clones that include the whole sequence of *Teratorn*; 3. Sequencing of BAC clones; 4. gene annotation. **(b)** Dot plots showing the alignment of one *Teratorn* copy (73I9; named from the ID of BAC clone) with the other five copies (13I17, 6L21, 11H24, 14A10, 85H23). The red and blue dots indicate that the corresponding residues of the two sequences match in the forward and reverse direction, respectively. Note that the synteny, including the position of repetitive region, is conserved among all copies. **(c)** Sequence comparison between one copy and the other five copies, visualized by VISTA. Purple and red indicate putative coding and non-coding regions, respectively. Arrows above the histogram indicate the position of genes in 73I9 copy.

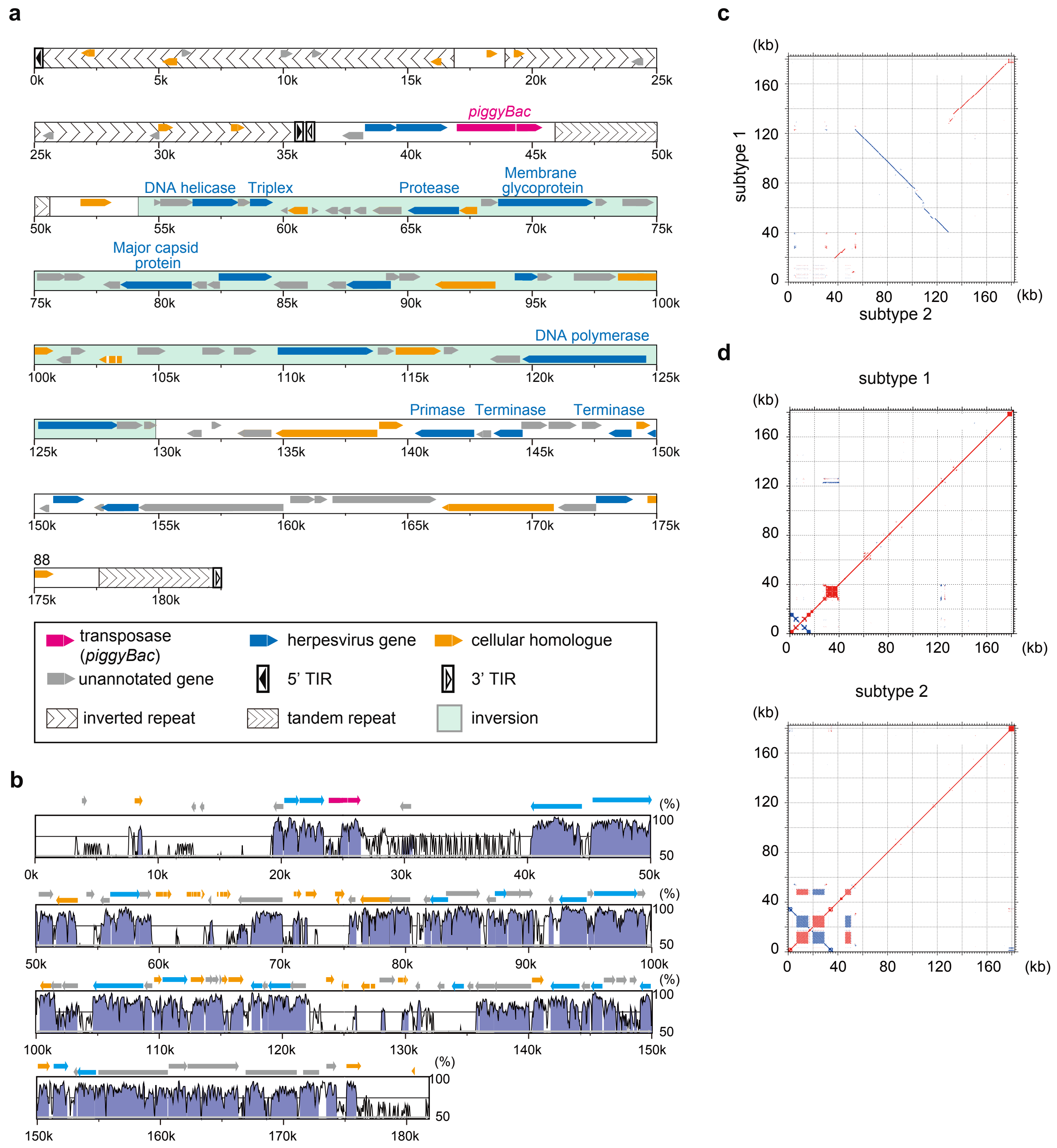

Supplementary Figure 2 l There are two subtypes of *Teratorn*. (a) Gene map of the consensus sequence of subtype 2 *Teratorn*. Predicted genes are classified into four categories depicted by colored arrowheads; magenta, *PiggyBac*-like transposase gene; blue, herpesvirus-like genes; yellow, cellular homologues; gray, unannotated genes. Terminal inverted repeats (TIR) of the *PiggyBac*-like transposon are depicted by boxed triangles. Light green region indicate a ~80-kb long inversion between subtype 1 and subtype 2. **(b)** Sequence comparison between *Teratorn* subtype 1 and subtype 2, visualized by VISTA. Blue and white regions indicate coding and non-coding regions, respectively. Arrows above the histogram indicate the position of genes inside Teratorn subtype 1. **(c)** A dot plot matrix showing the alignment of *Teratorn* subtype 1 and subtype 2. Note that synteny is almost conserved except for a ~80-kb long inversion in the middle. **(d)** Dot plots showing the alignment of each *Teratorn* subtype with itself. Note that the position of inverted repeat and tandem repeats are common between the two subtypes.

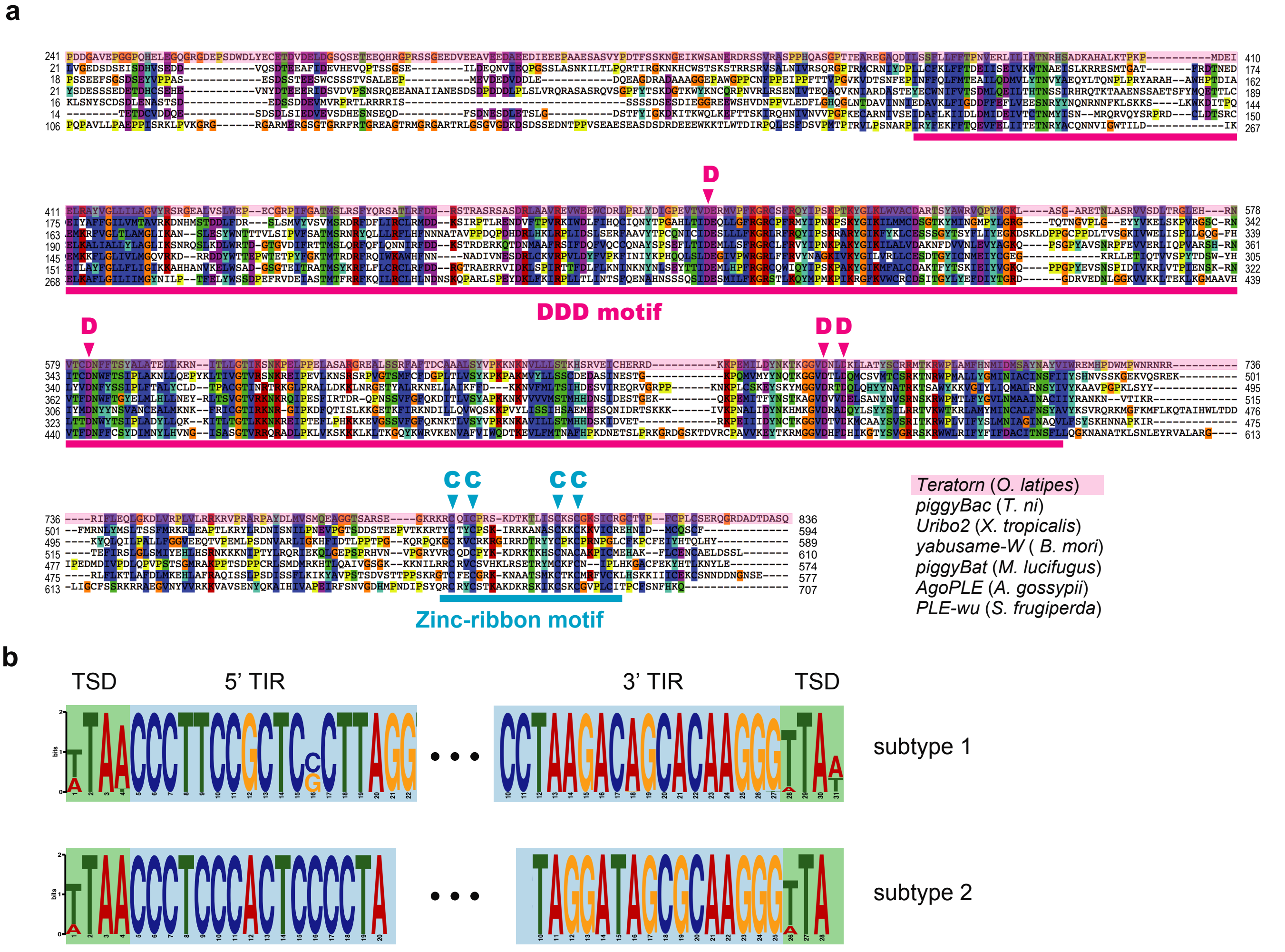

Supplementary Figure 3 | *Teratorn* belongs to the *piggyBac* superfamily. (a) Alignment of partial amino acid sequences of several active *piggyBac* superfamily transposase genes. Note the conservation of the four aspartic acid residues, which form the core of the 'DDD motif' and are required for the transposition reaction of *piggyBac* (magenta arrowheads). In addition, four cysteine residues at the C termini, which form the core of Zinc-ribbon motif with unknown function, are also conserved (blue arrowheads). **(b)** Consensus sequences of TIR and TSD of all *Teratorn* copies in the genome of the Hd-rR inbred strain, displayed by MEME. Note that sequence composition of TIR and TSD follow the rule of *piggyBac* superfamily; TIR length ranges from 12 to 19bp beginning with the "CCYT" motif, and targets TTAA.

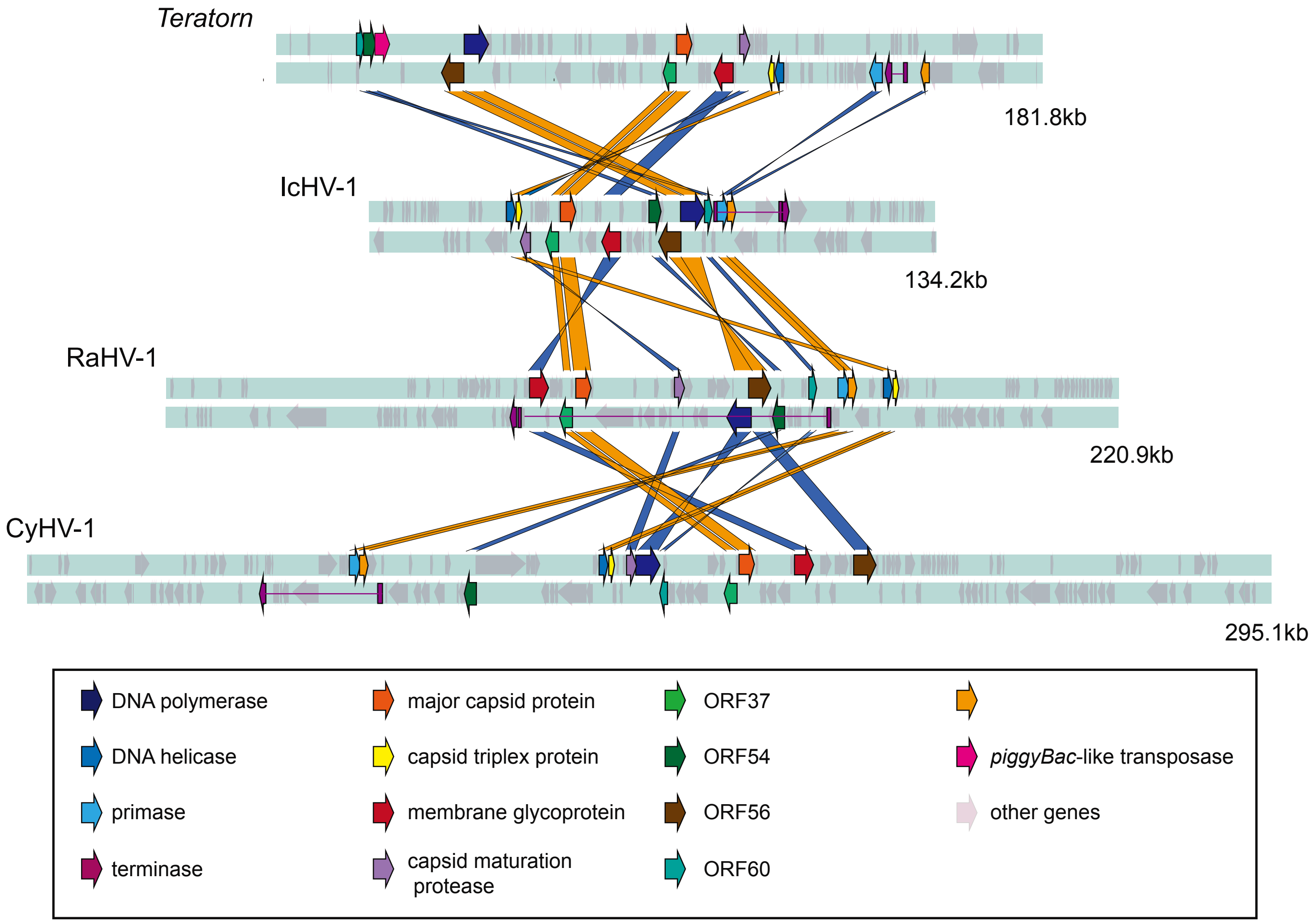

Supplementary Figure 4 l Genomic comparison of *Teratorn* and alloherpesvirus species. ORFs inside three alloherpesvirus species and *Teratorn* are drawn by arrows. Thirteen core genes indicated by colored arrows are connected by colored bars across different herpesvirus genomes (S. van Beurden and M. Engelsma, 2012[24]). Note that there are some conserved gene blocks among *Teratorn* and alloherpesvirus species (e.g. capsid triplex protein and DNA helicase, major capsid protein and ORF37, DNA polymerase and ORF56, primase and ORF64, orange bars), indicating that *Teratorn* belongs to the family *Alloherpesviridae*.

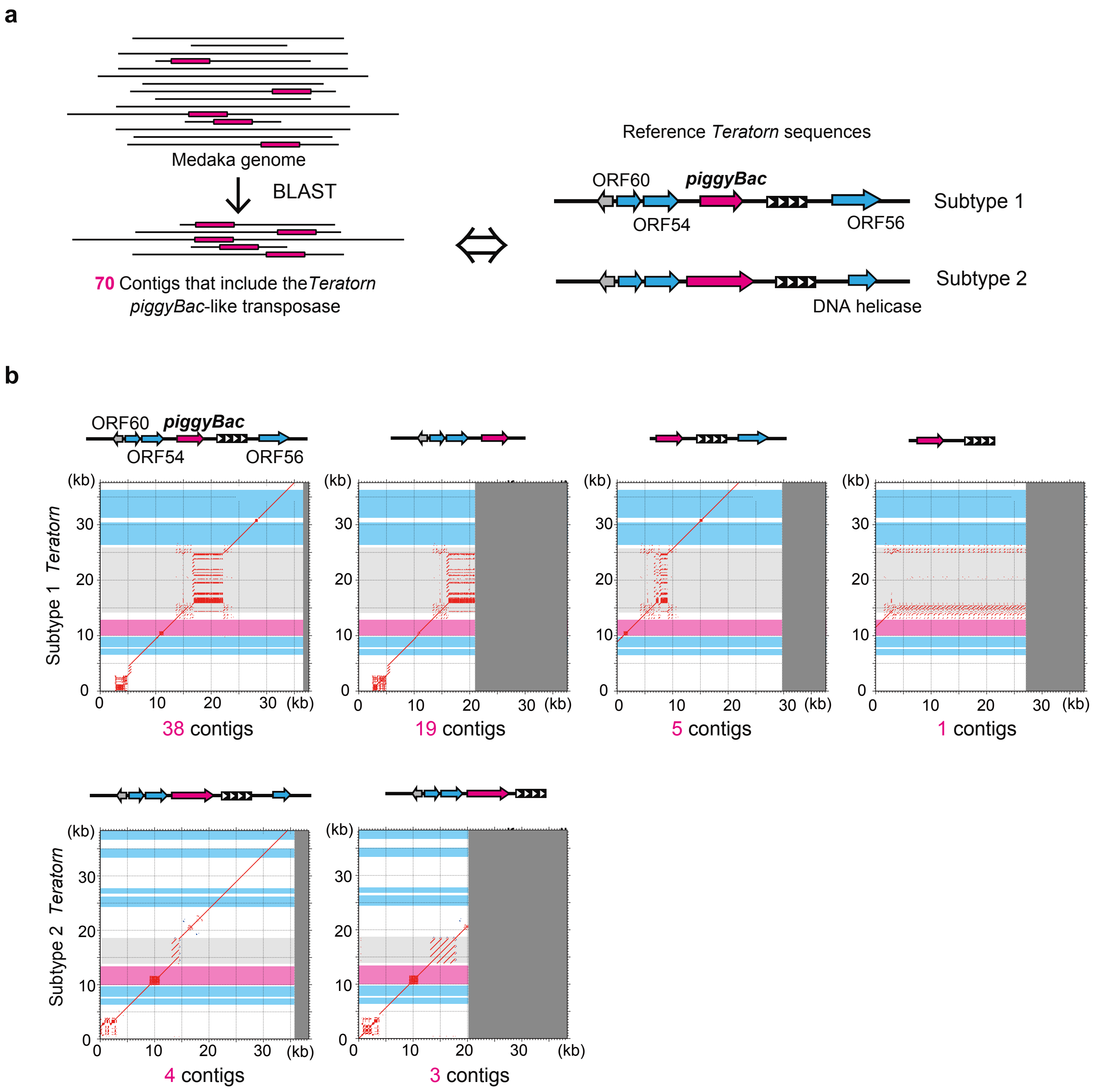

Supplementary Figure 5 | All of the *piggyBac*-like transposase copies are adjacent to the herpesvirus genes in the medaka genome. (a) The procedure of screening all contigs that include the *piggyBac*-like transposase gene of *Teratorn*. First, all contigs that contain the transposase gene were screened from the medaka draft genome by blastn. For all contigs obtained, genomic neighborhoods around the transposase genes were tested by displaying alignment with the reference *Teratorn* sequence. **(b)** Dot plots showing the alignment of the reference sequence of *Teratorn* with the contigs screened from the medaka draft genome. Magenta, cyan and light gray indicate the coding region of the *piggyBac*-like transposase gene, herpesvirus genes and tandem repeats, respectively.

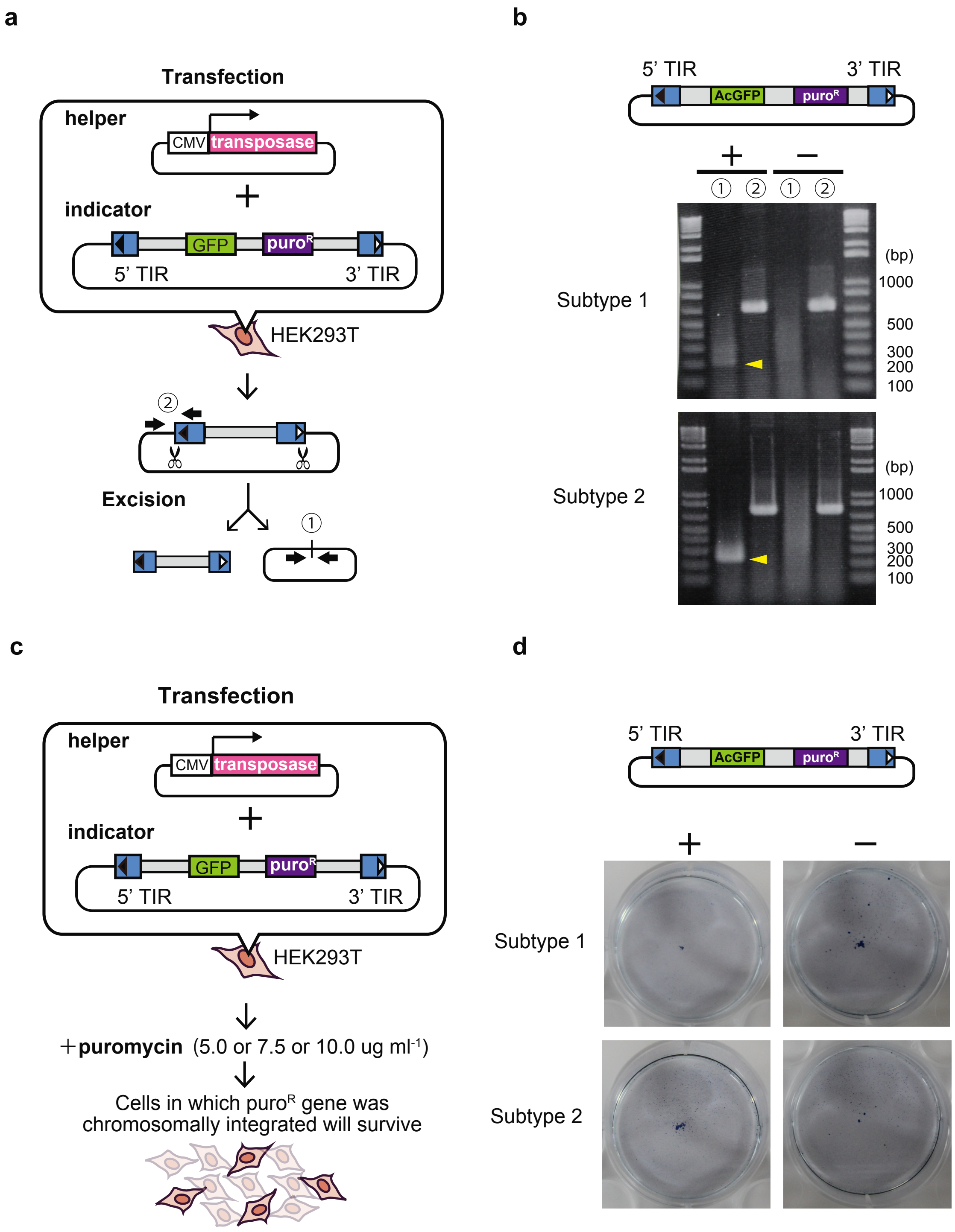

Supplementary Figure 6 | Transposition assay using indicator plasmid that doesn't contain internal TIRs. **(a)** A schematic of the excision assay. In the helper plasmid, *Teratorn* transposase gene was expressed under the CMV promoter. In the indicator plasmid, a GFP reporter and a puromycin-resistant gene were flanked by the 5' and 3' TIR. Note that internal TIRs were not included, in contrast to the experiment shown in Figure 3. Transposition activity was examined by co-transfection of those two plasmids into HEK293T cells, followed by PCR-based detection of transposon cassette excision from the indicator plasmid. Thick arrows indicate primer pairs used for the excision assay. **(b)** Results of the excision assay in the absence of internal TIR. 5' and 3' TIRs are sufficient for excision reaction. **(c)** Schematic of the integration assay. In this assay, long-term chemical selection was carried out following plasmid transfection to screen transgenic cell lines. **(d)** Results of the integration assay. Without internal TIRs, chromosomal integration didn't occur even in the presence of transposase.

Subtype 1

	flanking region	TSD	*Teratorn*	TSD	flanking region
indicator	TTTATCTGAT	TTAA	CCCTTCCGCT·····AGCACAAGGG	TTAT	AGACTTTGCA
1	TTTATCTGAT	TTAA	---------- ----------	----	AGACTTTGCA
2	TTTATCTGAT	TTAA	---------- ----------	----	AGACTTTGCA
3	TTTATCTGAT	TTAA	---------- ----------	----	AGACTTTGCA
4	TTTATCTGAT	TTAA	---------- ----------	----	AGACTTTGCA
5	TTTATCTGAT	TTAA	---------- ----------	----	AGACTTTGCA
6	TTTATCTGAT	TTAA	---------- ----------	-TAT	AGACTTTGCA
7	TTTATCTGAT	----	---------- ----------	TTAT	AGACTTTGCA
8	TTTATCTGAT	----	---------- ----------	TTAT	AGACTTTGCA
9	TTTATCTGAT	----	---------- ----------	TTAT	AGACTTTGCA
10	TTTATCTGAT	----	---------- ----------	TTAT	AGACTTTGCA
11	TTTATCTGAT	----	---------- ----------	TTAT	AGACTTTGCA
12	TTTATCTGAT	----	---------- ----------	TTAT	AGACTTTGCA
13	TTTATCTGAT	----	---------- ----------	TTAT	AGACTTTGCA
14	TTTATCTGAT	----	---------- ----------	TTAT	AGACTTTGCA
15	TTTATCTGAT	----	---------- ----------	TTAT	AGACTTTGCA
16	TTTATCTGAT	----	---------- ----------	TTAT	AGACTTTGCA
17	TTTATCTGAT	----	---------- ----------	TTAT	AGACTTTGCA
18	TTTATCTGAT	----	---------- ----------	TTAT	AGACTTTGCA
19	TTTATCTGAT	----	---------- ----------	TTAT	AGACTTTGCA
20	TTTATCTGAT	----	---------- ----------	TTAT	AGACTTTGCA
21	TTTATCTGAT	----	---------- ----------	TTAT	AGACTTTGCA
22	TTTATCTGAT	----	---------- ----------	TTAT	AGACTTTGCA
23	TTTATCTGAT	----	---------- ----------	TTAT	AGACTTTGCA
24	TTTATCTGAT	----	---------- ----------	TTAT	AGACTTTGCA
25	TTTATCTGAT	----	---------- ----------	TTAT	AGACTTTGCA
26	TTTATCTGAT	----	---------- ----------	TTAT	AGACTTTGCA
27	TTTATCTGAT	----	---------- ----------	TTAT	AGACTTTGCA
28	TTTATCTGAT	TTAA	---------- ----------	---T	AGACTTTGCA
29	TTTATCTGAT	----	---------- ---------A	TTAT	AGACTTTGCC

Subtype 2

	flanking region	TSD	*Teratorn*	TSD	flanking region
indicator	CAGAAGCTCA	TTAA	CCCTCCCACT·····AGCGCAAGGG	TTAT	TGAATTAAAA
1	CAGAAGCTCA	TTAA	---------- ----------	----	TGAATTAAAA
2	CAGAAGCTCA	----	---------- ----------	TTAT	TGAATTAAAA
3	CAGAAGCTCA	----	---------- ----------	TTAT	TGAATTAAAA
4	CAGAAGCTCA	----	---------- ----------	TTAT	TGAATTAAAA
5	CAGAAGCTCA	----	---------- ----------	TTAT	TGAATTAAAA
6	CAGAAGCTCA	----	---------- ----------	TTAT	TGAATTAAAA
7	CAGAAGCTCA	----	---------- ----------	TTAT	TGAATTAAAA
8	CAGAAGCTCA	----	---------- ----------	TTAT	TGAATTAAAA
9	CAGAAGCTCA	----	---------- ----------	TTAT	TGAATTAAAA
10	CAGAAGCTCA	----	---------- ----------	TTAT	TGAATTAAAA
11	CAGAAGCTCA	----	---------- ----------	TTAT	TGAATTAAAA
12	CAGAAGCTCA	----	---------- ----------	TTAT	TGAATTAAAA
13	CAGAAGCTCA	----	---------- ----------	TTAT	TGAATTAAAA
14	CAGAAGCTCA	----	---------- ----------	TTAT	TGAATTAAAA
15	CAGAAGCTCA	----	---------- ----------	TTAT	TGAATTAAAA
16	CAGAAGCTCA	----	---------- ----------	TTAT	TGAATTAAAA
17	CAGAAGCTCA	----	---------- ----------	TTAT	TGAATTAAAA
18	CAGAAGCTCA	----	---------- ----------	TTAT	TGAATTAAAA
19	CAGAAGCTC-	----	---------- ----------	TTAT	TGAATTAAAA
20	CAGAAGCTCA	TTAA	---------- ----------	TTAT	TGAATTAAAA
21	CAGAAGCTCA	TTAA	---------- ----------	TTAT	TGAATTAAAA
22	CAGAAGCTCA	TTA-	---------- ----------	-TAT	TGAATTAAAA
23	CAGAAGCTCA	TTA-	---------- ----------	----	----TTAAAA

Supplementary Figure 7 I Precise excision of *Teratorn* transposon cassette from the indicator plasmid. Nucleotide sequences of PCR products obtained from the excision assay, as described in Figure 3b. The top line indicates the sequence of the indicator plasmid, and the following 29 (subtype 1) and 23 (subtype 2) lines indicate the sequences of individual subcloned PCR products. Note that the excision reaction occurred precisely, at high frequency (subtype 1, 26/29 clones; subtype 2, 18/23 clones), similar to other *piggyBac* superfamily DNA transposons[28]. TSD; target site duplication.

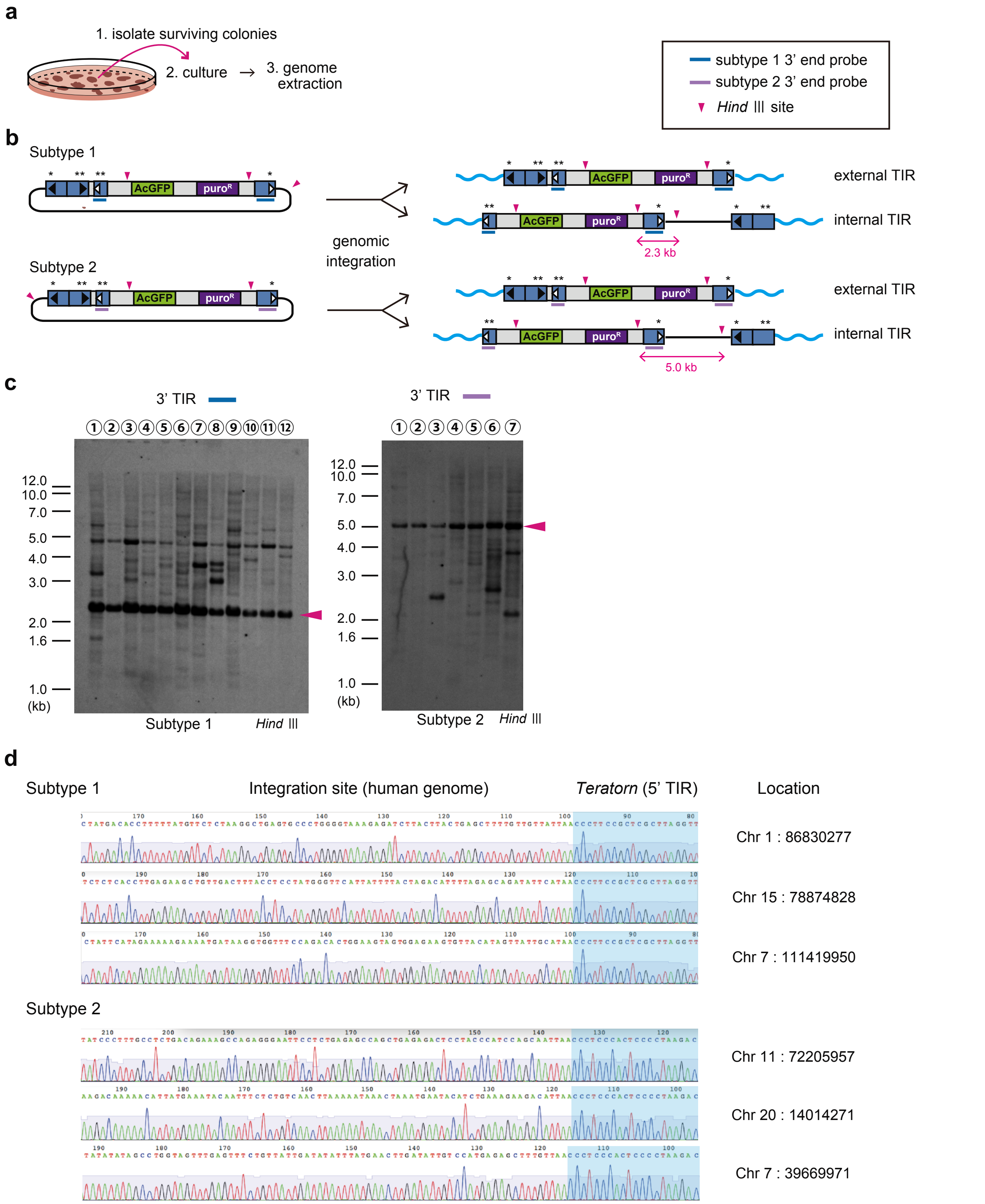

Supplementary Figure 8 I Confirmation of in vitro integration of *Teratorn* transposon cassette into HEK293T cells. (a) Isolation method of genomic DNA from cell colonies that survived puromycin selection. **(b)** Possible way of chromosomal integration of the indicator plasmids. Integration can be occurred via 1) external TIRs (upper) and/or internal TIRs (lower). Blue and purple bars indicate the position of the target sequences for southern hybridization (blue, subtype 1; purple, subtype 2). Magenta arrowheads indicate *Hind*III sites. **(c)** Southern blotting of genomic DNA of surviving colonies of HEK293T cells, using 3' terminal sequences as hybridization probes. Numbers above the lanes indicate the genomic DNA of individual colonies. Although the band pattern was different among individual clones, there is a common band (arrowheads) which size corresponds to the DNA fragment formed by *Hind*III digestion (depicted as double-headed arrows in b), suggesting that transposition via internal TIRs took place (as in b). **(d)** Examples of insertion sites of *Teratorn* transposon cassette in the genome of surviving HEK293T cell colonies. The blue region indicates the terminal sequence of *Teratorn*.

a

b

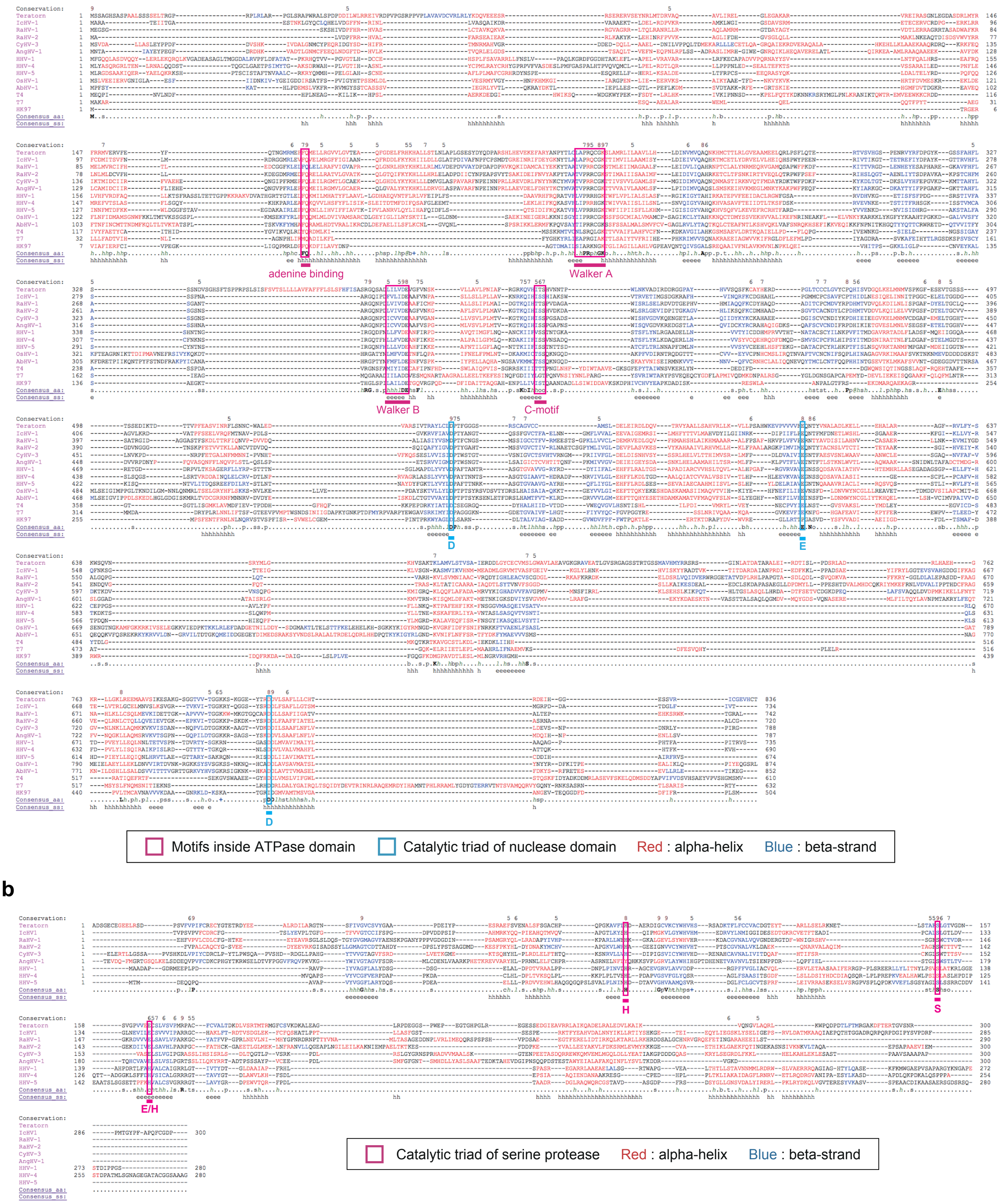

Supplementary Figure 9 | Catalytic residues of DNA packaging terminase and capsid maturation protease are conserved in *Teratorn*. (a) Multiple alignment of the full-length amino acid sequences of DNA packaging terminase gene, constructed by PROMALS3D[61] (*Teratorn*, magenta; *Alloherpesviridae*, blue; *Herpesviridae*, yellow; *Malacoherpesviridae*, black; bacteriophages, green). Catalytic center motifs are depicted by magenta (ATPase domain) and cyan (nuclease domain), respectively[30,31]. Note the sequence conservation at catalytic centers in *Teratorn*. **(b)** Multiple alignment of partial sequences of capsid maturation protease gene (*Teratorn*, magenta; *Alloherpesviridae*, blue; *Herpesviridae*, yellow). Note the sequence conservation of catalytic triads (His-Ser-His/Glu)[32].

a

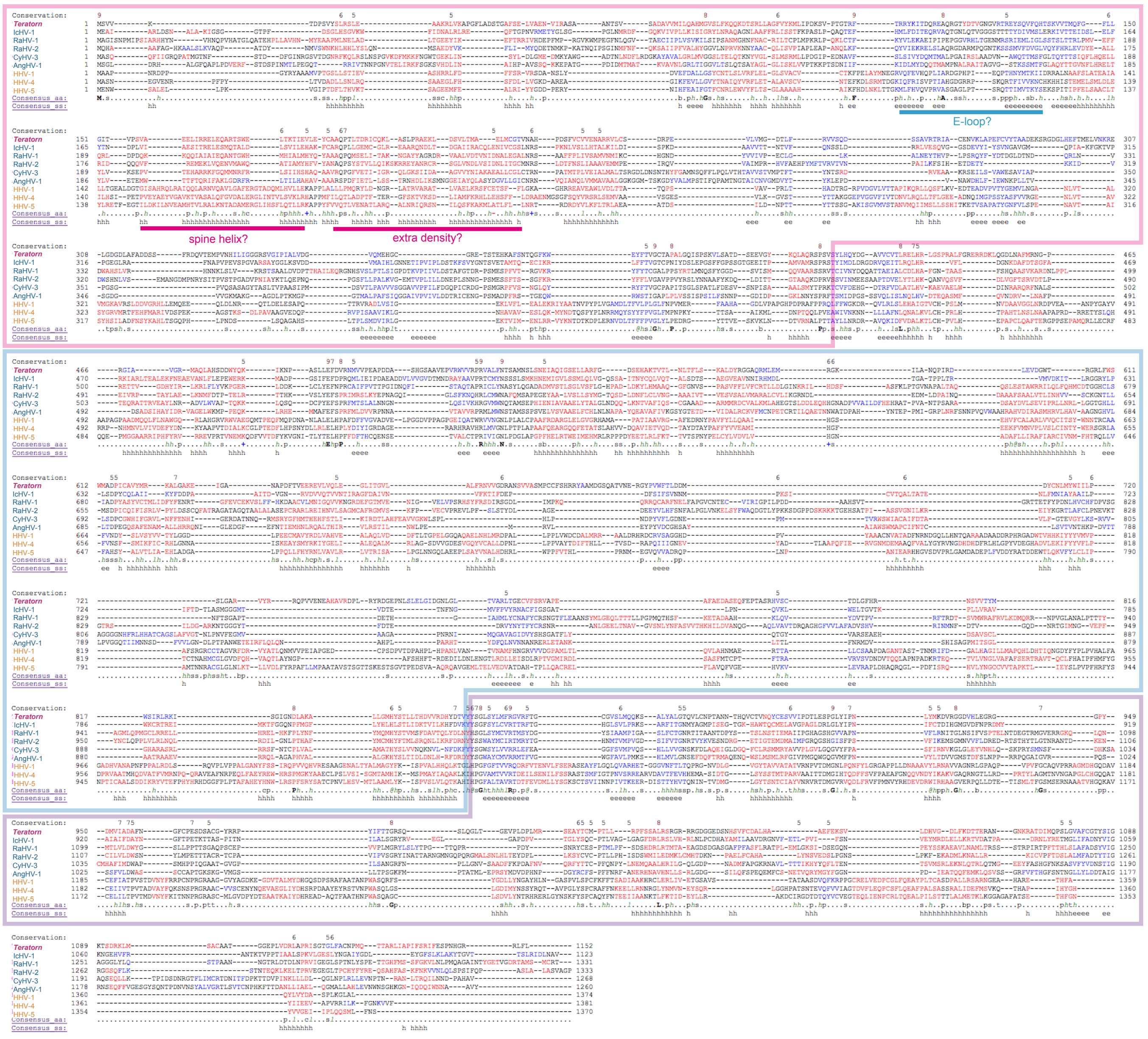

b

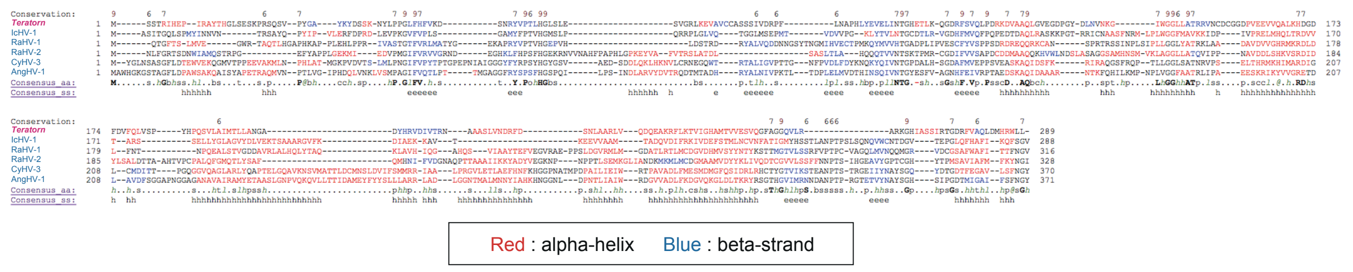

Supplementary Figure 10 l Similar secondary structure pattern of capsid proteins between herpesviruses and *Teratorn*. (a) Multiple alignment of the full-length amino acid sequences of major capsid protein, constructed by PROMALS3D[61] (*Teratorn* ,magenta; *Alloherpesviridae*, blue; *Herpesviridae*, yellow). Predicted secondary structures are depicted as red (α-helix) and blue (ß-strand). Herpesvirus major capsid proteins are known to be subdivided into three domains; Floor domain, which faces at the lumen of the capsid (pink), Upper domain, which faces at the outer surface of the capsid (blue), and Middle domain, which locates in the middle(purple)[33]. Although sequence similarity was low, similar pattern of secondary structures was observed. **(b)** Multiple alignment of the full-length sequence of subunit 2 capsid triplex protein of *Teratorn* (magenta) and alloherpesvirus species (blue). Note the sequence similarity among them.

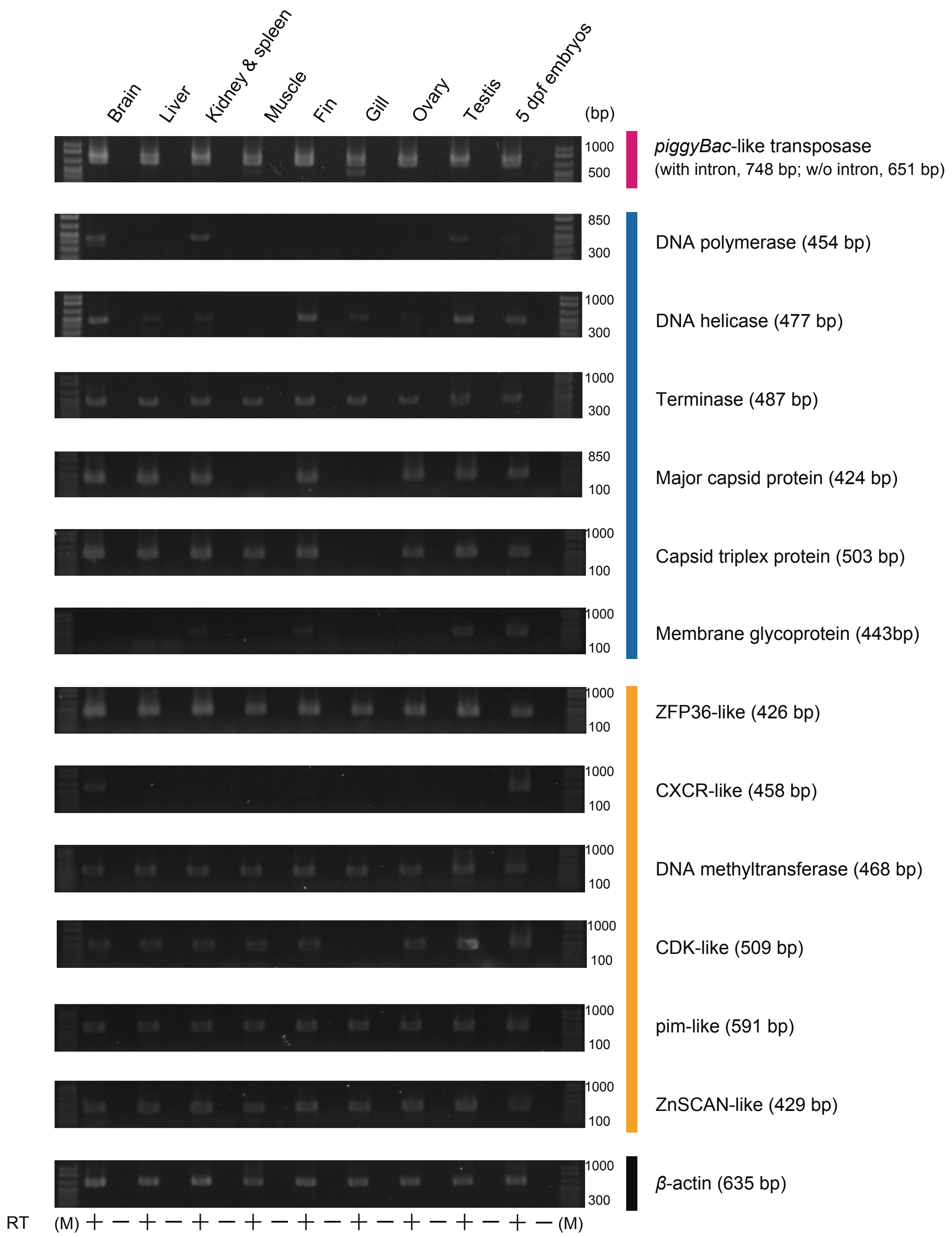

Supplementary Figure 11 l Expression of *Teratorn* genes in several medaka tissues. RT-PCR of *Teratorn* genes in several adult medaka tissues and 5 dpf medaka embryos. “+” and “–” indicate whether the reverse-transcription reaction was carried out or not, respectively. PCR amplification was repeated for 40 cycles.

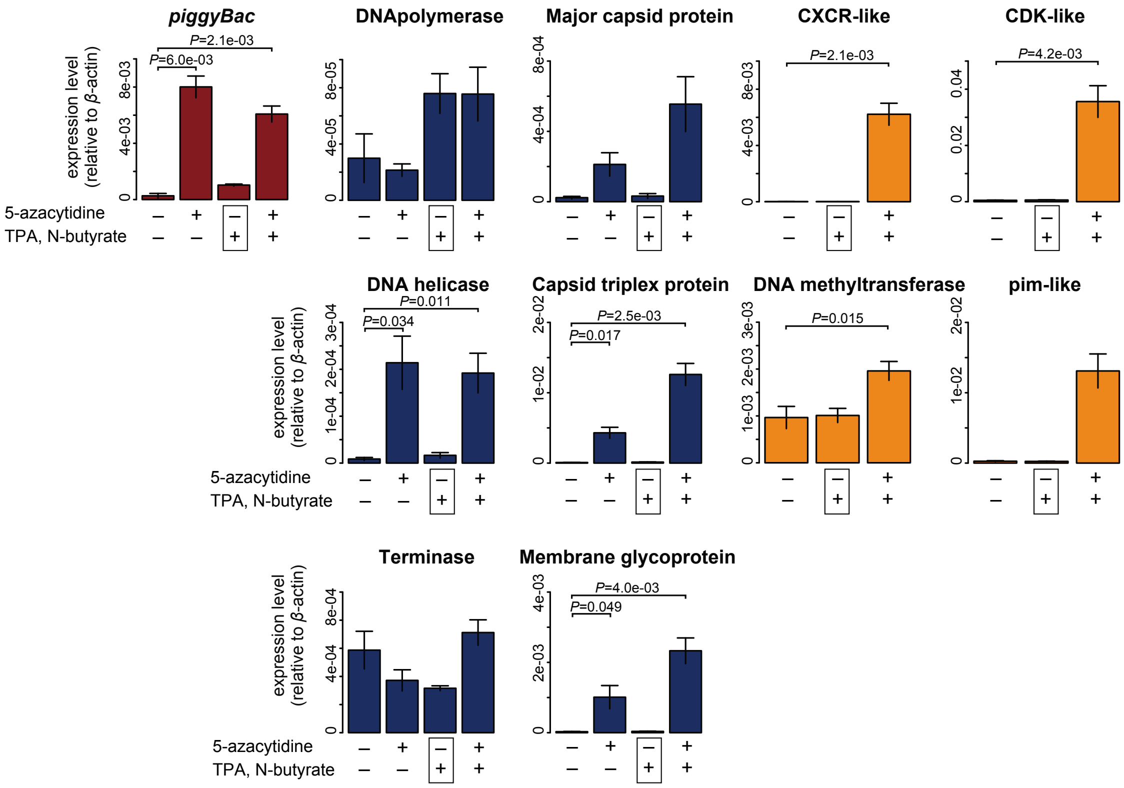

Supplementary Figure 12 | TPA and N-butyrate alone don't reactivate *Teratorn* genes. qPCR analysis of *Teratorn* genes in medaka fibroblast cells administered with or without 2μM of 5-azacytidine, 3mM of N-butyrate and 500ng ml^{-1} of 12-*O*-Tetradecanoylphorbol 13-acetate (TPA), same as the results presented in Fig. 4c. "+" and "–" indicate that each chemical was administered or not. The value indicates the ratio of molar concentration relative to *β*-actin. Note that administration of TPA and N-butyrate didn't reactivate *Teratorn* genes in the absence of 5-azacytidine. Statistical significance was tested by one-sided Welch Two Sample t-test. Error bars indicate s.e.m. Numbers of replicates are as follows; n = 3 for no chemical treatment, n = 3 for 5-azacytidine treatment, n = 4 for TPA and N-butyrate treatment, n = 4 for 5-azacytidine, TPA and N-butyrate treatment.

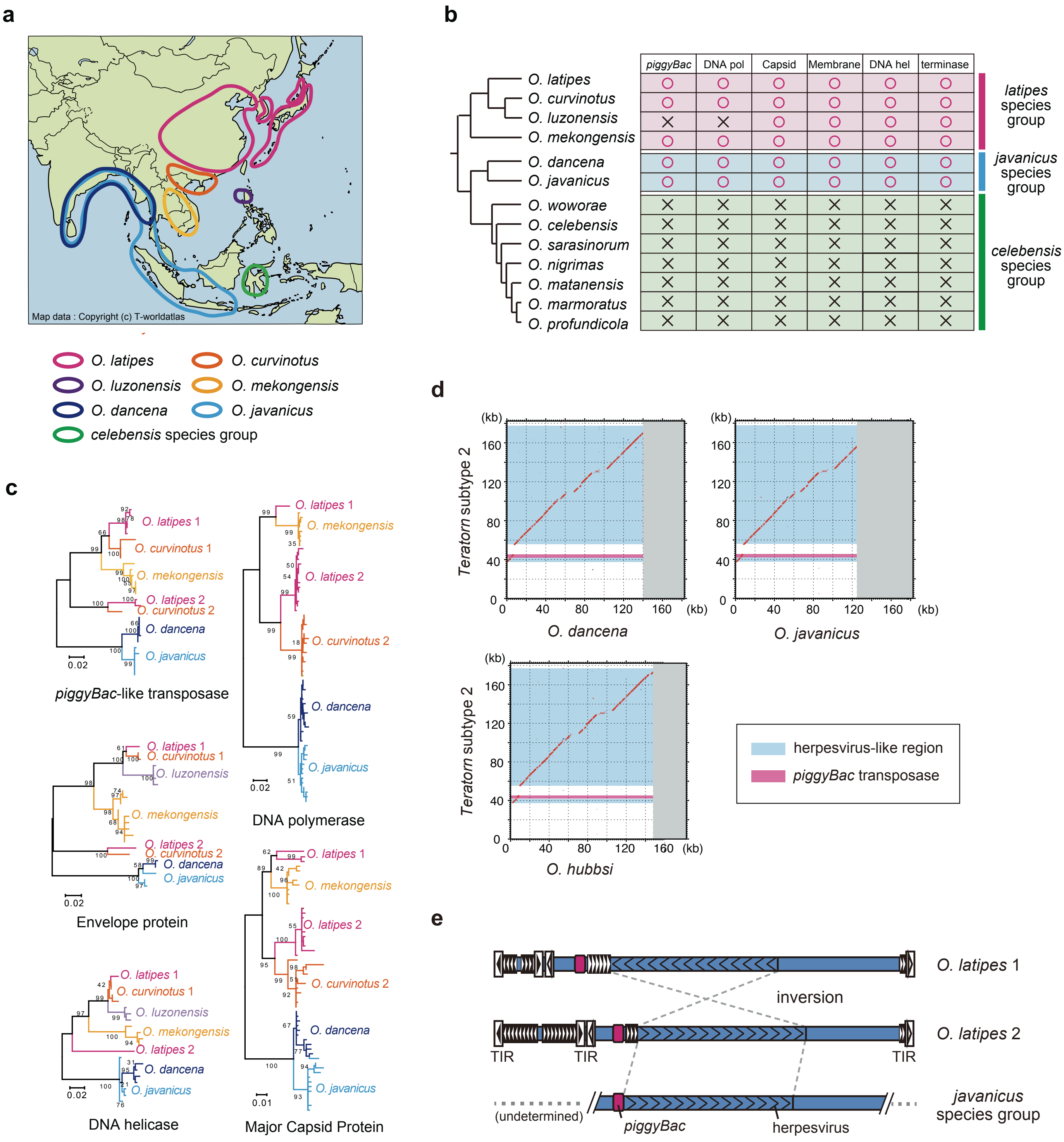

Supplementary Figure 13 | Widespread colonization of *Teratorn* in *Oryzias* genus. (a) Geographic distribution of medaka related species. **(b)** Results of PCR screening of six *Teratorn* genes in 13 medaka related species. Phylogenetic relationships of medaka related species used in this analysis are depicted on the left[39]. Note that *Teratorn* genes are detected in *latipes* and *javanicus* species group but not in *celebensis* species group. **(C)** Maximum-likelihood trees based on the sequences of subcloned PCR products of *Teratorn* genes from each medaka related species. Note that the topology of the phylogenetic trees of each gene are almost the same as that of host species, except for the existence of two subtypes of *Teratorn* in *latipes* species group. The scale bar represents the number of substitutions per site. **(d)** Dot plots showing the alignment of *Teratorn* in medaka species of *javanicus* species group with subtype 2 *Teratorn* in *O. latipes*. *piggyBac*-like transposase gene and herpesvirus-like region are shown in magenta and cyan, respectively. **(e)** Comparison of the structure of *Teratorn* in *Oryzias* genus. Note that gene synteny, including the position of the *piggyBac* transposase gene, is conserved in the *Oryzias* genus.

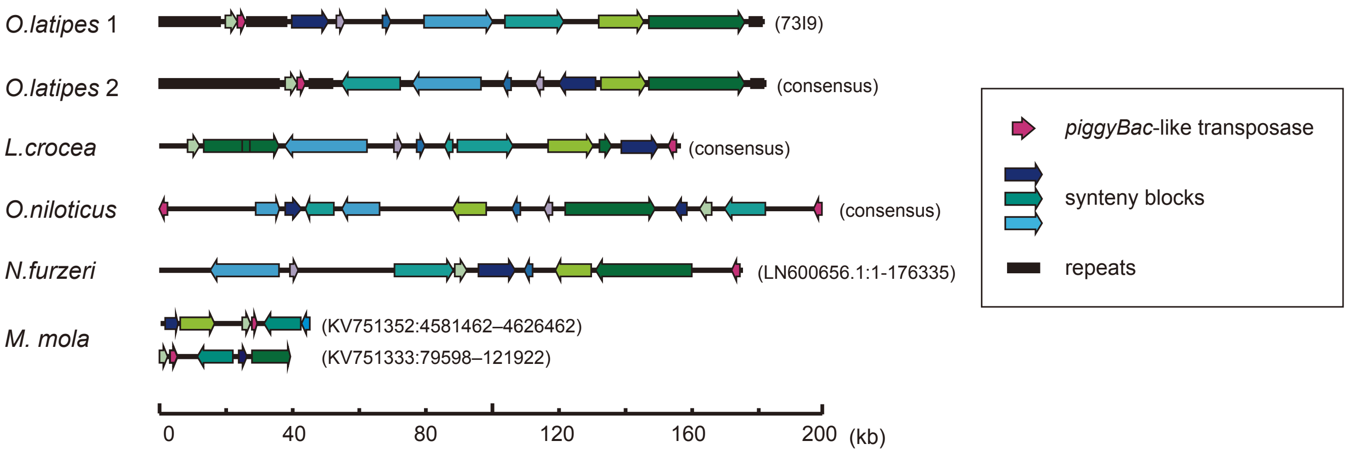

Supplementary Figure 14 l Identification of *Teratorn*-like elements in teleosts. Structures of *Teratorn*-like elements in several teleost fish species, in which *piggyBac*-herpesvirus fusion was suggested. Those sequences were (1) extracted from contigs or scaffolds (*N. furzeri* and *M. mola*) or (2) reconstructed by conjugating several contigs (*L. crocea* and *O. niloticus*). Conserved synteny blocks are depicted by arrows of the same colors. Magenta arrows indicate *piggyBac*-like transposase.

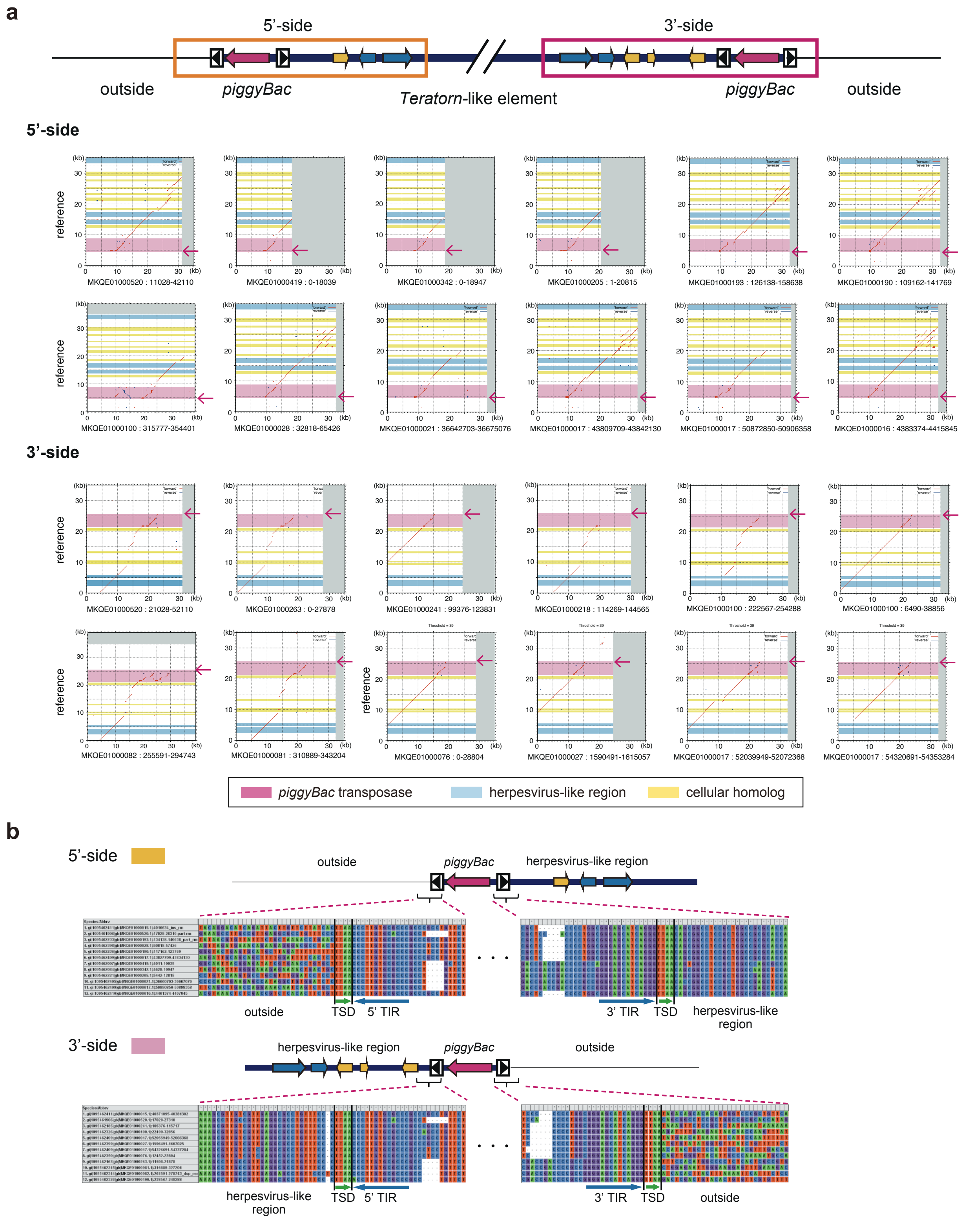

Supplementary Figure 15 | TIR of *piggyBac* transposon is located at the boundary of *Teratorn*-like elements in nile tilapia. (a) Dot plots showing the alignment of terminal region of one copy of *Teratorn*-like element (5' end, MKQE01000015.1:40163261–4094943 (the copy-specific insertion (40171174–40177855) was removed); 3' end, MKQE01000015.1:40353261–40388260) and other copies obtained by blast. Magenta, blue and yellow region indicate the *piggyBac*, herpesvirus genes and cellular homologues, respectively. Note that sequence conservation was seen only interior to the *piggyBac* transposon, and the boundary corresponds to the position of terminal inverted repeat (magenta arrows). **(b)** Multiple alignments of *piggyBac* transposon copies adjacent to the herpesvirus-like region (5' side, 12 loci; 3' side, 12 loci). Terminal region of *piggyBac* transposon is displayed. Note that sequences are almost identical from the 5' TIR (terminal inverted repeat) of the 5'–side *piggyBac* to the 3' TIR of the 3'–side *piggyBac*, while no sequence similarity is found outside, suggesting the transposition of *Teratorn*-like element via *piggyBac* of both ends. TSD: target site duplication.

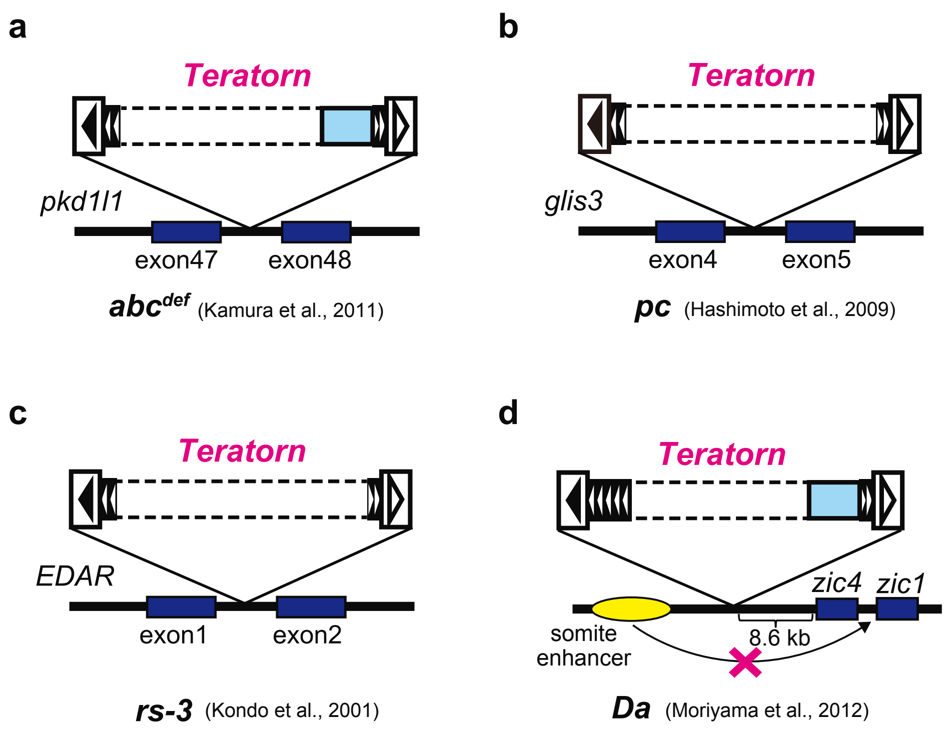

Supplementary Figure 16 l Medaka spontaneous mutants caused by *Teratorn* insertion. (a) *abc^{def}* mutant (defect in left-right axis formation)[43]. **(b)** *pc* mutant (polycystic kidney disease)[42]. **(c)** *rs-3* mutant (loss of scales)[41]. **(d)** *Da* mutant (ventralization of dorsal trunk morphology)[12]. Solid and dotted boxes inside *Teratorn* indicate the sequence-determined and undetermined region, respectively.

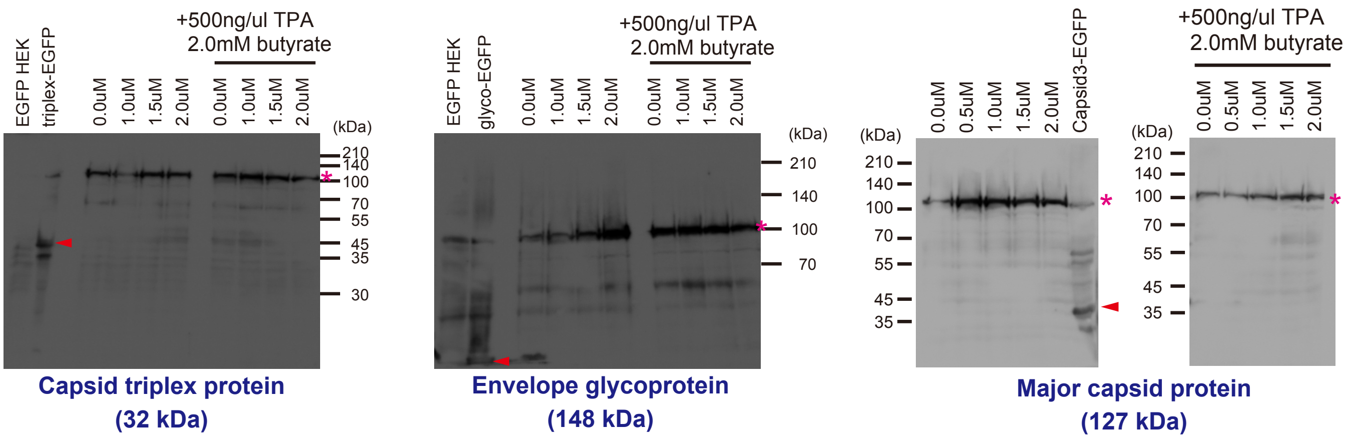

Supplementary Figure 17 | No virus structural protein production after administration of 5-azacytidine, TPA and N-butyrate in medaka fibroblasts. Western blot of the lysate of medaka fibroblasts administered with 5-azacytidine, TPA and N-butyrate for the presence of three herpesvirus structural proteins (capsid triplex protein, envelope glycoprotein and major capsid protein) of *Teratorn*. Arrowheads indicate the positive control; lysate of cells transfected with the plasmids that express the antigen protein fragment fused with GFP. Note that no clear signal was observed at the corresponding molecular weight for each gene. Asterisks indicate a non-specific signal.

Supplementary Tables

Supplementary Table 1 | Predicted ORFs inside *Teratorn* subtype 1

Gene	properties or putative functions	Evalue	start	end	direction	IcHV-1		CyHV-3		RaHV-1	
						ORF	Evalue	ORF	Evalue	ORF	Evalue
ORF1			3689	4048	+						
ORF2	myogenic factor MyoD2 (O. niloticus)	0.005	8022	8630	+						
ORF3			12667	13026	-						
ORF4			13388	13702	-						
ORF5			19344	20096	-						
**ORF6**	**ORF60**	**9.00E-22**	**20228**	**21406**	**+**	**ORF60**	**9.00E-22**	**ORF80L**	**6.00E-04**	**ORF84**	**5.00E-08**
**ORF7**	**ORF54**	**6.00E-05**	**21471**	**23450**	**+**	**ORF54**	**6.00E-05**	**ORF61**	**-**	**ORF75**	**-**
**ORF8**	***piggyBac*-like transposase**	**0**	**23875**	**26466**	**+**						
ORF9			29685	30569	-						
**ORF10**	**ORF56**	**4.00E-41**	**40287**	**44351**	**-**	**ORF56**	**1.00E-40**	**ORF107**	**2.00E-18**	**ORF73**	**2.00E-33**
**ORF11**	**DNA polymerase**	**2.00E-111**	**45181**	**50085**	**+**	**ORF57**	**2.00E-111**	**ORF79**	**7.00E-57**	**ORF72**	**6.00E-73**
ORF12			50203	51330	+						
ORF13	OTU-like cysteine protease domain (C. semilaevis)	2.00E-35	51603	53297	-						
ORF14			54098	54694	+						
ORF15			55227	56054	-						
ORF16	ORF73_protein kinase (IcHV-1)	4.00E-09	56053	58443	+	ORF73	4.00E-09				
ORF17			58410	59327	+						
ORF18	CD276 antigen-like (O. latipes)	8.00E-32	59157	61143	+						
ORF19	CD276 antigen-like (H. burtoni)	1.00E-19	62103	63705	+						
ORF20			64024	64353	-						
ORF21	HERV-H LTR-associating protein 2-like (P. nyererei)	1.00E-17	64751	65805	+						
ORF22			66376	70044	-						
ORF23	mcl-1 (O. mykiss)	1.00E-19	70917	71457	+						
ORF24	CDK2 (L. crocea)	2.00E-80	71862	72749	+						
ORF25	zinc finger protein 36, C3H1 type-like 2 (S. partitus)	2.00E-20	74275	75075	+						
ORF26	integrase / recombinase (Vibrio splendidus)	0.011	74362	74691	-						
ORF27			75467	76012	-						
ORF28	CAP-Gly domain containing linker protein 1-like (S. partitus)	0.02	76476	79142	-						
ORF29			78757	80472	-						
ORF30			81622	82077	-						
ORF31	ORF44	8.00E-18	82121	83569	-	ORF44	8.00E-18			ORF59	3.00E-05
ORF32			83417	86155	+						
ORF33			86700	87455	-						
ORF34			87418	88338	+						
ORF35	ORF34	7.00E-50	88079	89569	+	ORF34	4.00E-43			ORF49	5.00E-31
ORF36			89566	90429	+						
ORF37			91720	92091	-						
**ORF38**	**ORF37**	**6.00E-08**	**92640**	**94820**	**-**	**ORF37**	**3.00E-04**	**ORF90L**	**0.53**	**ORF52**	**-**
ORF39			94944	95378	+						
**ORF40**	**Major capsid protein**	**1.00E-22**	**95469**	**98927**	**+**	**ORF39**	**8.00E-21**	**ORF92**	**0.059**	**ORF54**	**2.00E-10**
ORF41			99000	99578	+						
ORF42	diguanylate cyclase (Firmicutes bacterium)	0.024	100296	101141	-						
ORF43			101152	101997	-						
ORF44			102065	103342	-						
**ORF45**	**Membrane glycoprotein**	**2.00E-89**	**104618**	**108688**	**-**	**ORF46**	**6.00E-71**	**ORF99**	**1.00E-07**	**ORF46**	**1.00E-08**
ORF46			108803	109411	-						
ORF47	myopalladin, partial, (O. latipes)	4.00E-20	109609	110190	+						
**ORF48**	**Capsid maturation protease**	**5.00E-13**	**110276**	**112294**	**+**	**ORF28**	**1.00E-08**	**ORF78**	**-**	**ORF63**	**0.006**
ORF49	unnamed protein (O. mykiss)	5.00E-29	112609	113673	+						
ORF50			113800	114390	+						
ORF51			114397	114924	+						
ORF52			114956	115129	+						
ORF53	thiopurine S-methyltransferase (Pseudomonas fluorescens)	0.027	115138	115542	+						
ORF54	zinc finger BED domain-containing protein 4 (L. crocea)	2.00E-27	115680	116837	+						

Supplementary Table 1 Continued

Gene	properties or putative functions	Evalue	start	end	direction	IcHV-1 ORF	IcHV-1 Evalue	CyHV-3 ORF	CyHV-3 Evalue	RaHV-1 ORF	RaHV-1 Evalue
**ORF55**	**Capsid triplex protein subunit 2**	**4.00E-05**	**117519**	**118388**	**-**	**ORF27**	**1.00E-04**	**ORF72**	**-**	**ORF95**	**-**
ORF56			118445	118855	-						
**ORF57**	**DNA helicase (UL9 homolog)**	**2.00E-47**	**118921**	**120687**	**-**	**ORF25**	**7.00E-25**	**ORF71**	**2.00E-35**	**ORF93**	**3.00E-26**
ORF58			120719	121948	-						
ORF59	putative permease YjgP/YjgQ family protein (E. coli)	2.00E-12	123657	124283	+						
ORF60	zinc finger and SCAN domain-containing protein 29 (A. platyrhynchos)	2.00E-18	124919	125347	-						
ORF61	type-2 angiotensin II receptor-like cxcr (O. afer)	3.00E-13	126419	127532	-						
ORF62			127937	129121	+						
ORF63	75-interferon_induced_dsRNA_activated protein kinase (H. microstoma)	1.00E-07	129414	130175	+						
ORF64			130819	131172	-						
ORF65			132599	133129	-						
ORF66	ORF57R (CyHV-3)	5.00E-10	133776	134714	-			ORF57R	1.00E-10		
ORF67			135058	135489	-						
ORF68			135683	137212	-						
ORF69			137209	140187	-						
ORF70	DNA methyltransferase (Lymphocystis disease virus 1)	7.00E-07	140334	141221	+						
**ORF71**	**Primase**	**1.00E-14**	**141798**	**144128**	**-**	**ORF63**	**1.00E-11**	**ORF46**	**9.00E-04**	**ORF87**	**1.00E-08**
ORF72			144317	144970	-						
**ORF73**	**DNA packaging terminase subunit 1**	**4.00E-41**	**145083**	**146207**	**-**	**ORF62**	**4.00E-41**	**ORF33**	**9.00E-27**	**ORF42**	**1.00E-21**
ORF74			146149	147051	+						
ORF75			147148	147954	+						
ORF76			148268	148777	+						
**ORF77**	**DNA packaging terminase subunit 1**	**2.00E-12**	**149071**	**149904**	**-**	**ORF62**	**1.00E-09**	**ORF33**	**5.00E-05**		
ORF78	cbp_p300-interacting transactivator (L. crocea)	9.00E-90	149947	150927	+						
ORF79	ORF70 (RaHV-2)	0.69	151279	152451	+						
ORF80			152882	153208	-						
**ORF81**	**ORF64**	**9.00E-13**	**153189**	**154802**	**-**	**ORF64**	**9.00E-13**	**ORF47**	**-**	**ORF88**	**-**
ORF82			154685	160591	-						
ORF83			160679	162142	+						
ORF84			162226	166377	+						
ORF85			166751	171127	-						
ORF86			171416	172927	-						
ORF87			173599	174402	+						
ORF88	serine/threonine-protein kinase pim3 (A. mexicanus)	8.00E-76	175073	176215	+						
ORF89	chloride channel CLIC-like, partial (S. salar)	6.00E-07	180402	180593	-						

Supplementary Table 2 | Predicted ORFs inside *Teratorn* subtype 2

Gene	properties or putative functions	Evalue	start	end	direction	IcHV-1 ORF	IcHV-1 Evalue	CyHV-3 ORF	CyHV-3 Evalue	RaHV-1 ORF	RaHV-1 Evalue
ORF1L	HAF repeat protein (G. daltonii)	0.01	2018	2494	-						
ORF2L	signal-inducel proliferation-associated 1-like (S. formosus)	3.00E-10	5267	5791	-						
ORF3L			5790	6113	+						
ORF4L			10039	10425	+						
ORF5L			11281	11604	+						
ORF6L	RNA-directed DNA polymerase from jockey (L. crocea)	2.00E-08	16049	16435	-						
ORF7	RNA-directed DNA polymerase from jockey (A. queenslandica)	3.00E-11	18387	19124	+						
ORF6R	RNA-directed DNA polymerase from jockey (L. crocea)	2.00E-08	19368	19754	+						
ORF5R			24136	24522	-						
ORF4R			25378	25764	-						
ORF3R			29690	30013	-						
ORF2R	signal-inducel proliferation-associated 1-like (S. formosus)	3.00E-10	30012	30536	+						
ORF1R	HAF repeat protein (G. daltonii)	0.01	33288	33764	+						
ORF8			37404	38168	-						
**ORF9**	**ORF60**	**5.00E-23**	**38315**	**39529**	**+**	**ORF60**	**5.00E-23**	**ORF80L**	**0.002**	**ORF84**	**1.00E-05**
**ORF10**	**ORF54**	**0.004**	**39927**	**41522**	**+**	**ORF54**	**4.00E-03**	**ORF61**	**-**	**ORF75**	**-**
**ORF11**	***piggyBac*-like transposase**	**0.00E+00**	**41904**	**45337**	**+**						
ORF12	myogenic factor MyoD2 (C. mydas)	2.00E-09	51877	52896	+						
ORF13			53663	54127	+						
ORF14			54981	56315	+						
ORF15	**DNA helicase (UL9 homolog)**	**4.00E-47**	**56356**	**58137**	**+**	**ORF25**	**3.00E-25**	**ORF71R**	**7.00E-42**	**ORF93**	**1.00E-27**
ORF16			58213	58623	+						
ORF17	**Capsid triplex protein subunit 2**	**9.00E-05**	**58666**	**59535**	**+**	**ORF27**	**5.00E-04**	**ORF72**	**-**	**ORF95**	**-**
ORF18			59742	60131	-						
ORF19	zinc finger BED domain-containing protein 4 (L. crocea)	2.00E-22	60205	60621	-						
ORF20			60613	61062	+						
ORF21			61707	62165	-						
ORF22			62382	62909	-						
ORF23			62916	63506	-						
ORF24			63630	64706	-						
ORF25	**Capsid maturation protease**	**6.00E-12**	**65049**	**67133**	**-**	**ORF28**	**1.00E-09**	**ORF78**	**-**	**ORF63**	**8.00E-05**
ORF26	myopalladin, partial (O. latipes)	2.00E-09	67177	67761	-						
ORF27			67770	68567	+						
ORF28	**Membrane glycoprotein**	**1.00E-76**	**68678**	**72244**	**+**	**ORF46**	**4.00E-61**	**ORF99**	**3.00E-06**	**ORF46**	**3.00E-08**
ORF29			72308	72745	+						
ORF30			73699	75159	+						
ORF31			75261	76163	+						
ORF32			76281	77024	+						
ORF33			77868	78446	-						
ORF34	**Major capsid protein**	**2.00E-36**	**78524**	**81322**	**-**	**ORF39**	**2.00E-36**	**ORF92R**	**0.013**	**ORF54**	**2.00E-07**
ORF35			81282	81962	-						
ORF36			82046	82480	-						
ORF37	**ORF37**	**6.00E-07**	**82567**	**84762**	**+**	**ORF37**	**4.00E-04**	**ORF90L**	**1.90E-01**	**ORF52**	**-**
ORF38			84698	85987	-						
ORF39			86723	87553	-						
ORF40	ORF34	2.00E-45	87634	89127	-	ORF34	2.00E-36			ORF49	2.00E-26
ORF41			88886	89782	-						
ORF42			89745	90503	+						
ORF43	EH28 (L. crocea)	2.00E-112	91199	93949	-						
ORF44	ORF44	3.00E-20	94653	95249	+	ORF44	5.00E-18			ORF59	4.00E-08
ORF45			95298	95900	+						
ORF46			96733	98349	+						
ORF47	chromosome segregation ATPase domain		98647	100746	+						
ORF48			100966	101391	-						
ORF49			101552	102097	+						
ORF50	mcl-1 (P. reticulata)	5.00E-19	102609	103540	-						
ORF51			104244	106058	+						

Supplementary Table 2 Continued

Gene	properties or putative functions	Evalue	start	end	direc tion	IcHV-1		CyHV-3		RaHV-1	
						ORF	Evalue	ORF	Evalue	ORF	Evalue
ORF52			107323	107808	+						
ORF53			108960	109283	+						
ORF54	ORF73_protein-kinase (IcHV-1)	2.00E-08	109630	113034	-	ORF73	2.00E-08				
ORF55			113074	113862	+						
ORF56	OTU-like cysteine protease domain	8.00E-30	114609	115940	+						
ORF57			115912	116328	+						
ORF58			118409	119536	-						
ORF59	**DNA polymerase**	**1.00E-99**	**119679**	**124610**	**-**	**ORF57**	**1.00E-99**	**ORF79R**	**1.00E-53**	**ORF72**	**5.00E-75**
ORF60	**ORF56**	**2.00E-44**	**125229**	**128513**	**+**	**ORF56**	**8.00E-41**	**ORF107R**	**4.00E-18**	**ORF73**	**3.00E-34**
ORF61			128443	129324	+						
ORF62			129467	129901	+						
ORF63			131394	131708	-						
ORF64			132035	132493	+						
ORF65			133238	134752	-						
ORF66	tumor necrosis factor receptor 6B-like (L. crocea)	5.00E-04	134749	138765	-						
ORF67	DNA methyltransferase (Lymphocystic disease virus 1)	8.00E-08	138881	139774	+						
**ORF68**	**primase**	**8.00E-14**	**140358**	**142682**	**-**	**ORF63**	**1.00E-11**	**ORF46R**	**7.00E-06**	**ORF87**	**9.00E-07**
ORF69			142776	143324	-						
**ORF70**	**DNA packaging terminase subunit 1**	**2.00E-41**	**143537**	**144751**	**-**	**ORF62**	**2.00E-41**	**ORF33L**	**2.00E-27**	**ORF42**	**3.00E-21**
ORF71			144621	145595	+						
ORF72			145737	146765	+						
ORF73			147230	147760	+						
**ORF74**	**DNA packaging terminase subunit 1**	**1.00E-14**	**148139**	**148972**	**-**	**ORF62**	**1.00E-08**	**ORF33L**	**3.00E-04**	**ORF42**	**-**
ORF75	cbp_p300-interacting transactivator (L. crocea)	5.00E-85	149197	150045	+						
**ORF76**	**DNA packaging terminase subunit 1**	**4.00E-06**	**149968**	**150306**	**-**	**ORF62**	**4.00E-06**	**ORF33L**	**0.066**	**ORF42**	**9.00E-05**
ORF77			150567	150866	-						
ORF78	ORF70 (RaHV-2)	0.13	150869	151912	+						
ORF79			152462	152794	-						
**ORF80**	**ORF64**	**1.00E-14**	**152775**	**154400**	**-**	**ORF64**	**1.00E-14**	**ORF47**	**-**	**ORF88**	**-**
ORF81			154274	160384	-						
ORF82			160328	161767	+						
ORF83			161778	162113	+						
ORF84			162475	166134	+						
ORF85	sorbitol dehydrogenase (M. saccharolyticus)	0.007	166477	170865	-						
ORF86			171067	172581	-						
ORF87	ORF78 (IcHV-1)	0.13	172628	174052	+	ORF78	0.13				
ORF88	serine/threonine-protein kinase pim3 (S. salar)	4.00E-77	174720	175745	+						

Supplementary Table 3 | Variants inside coding region of subtype 1 *Teratorn*.

#GeneName	frameshift	stop_gained	inframe_del	inframe_ins	nonsynonymous	synonymous
1	0	0	0	0	0	0
2-MyoD2	0	0	0	0	17	8
3	0	0	0	0	0	0
4	0	0	0	0	0	0
5	0	0	0	0	8	10
6-ORF60	1	0	0	0	32	24
7-ORF54	0	4	0	1	78	46
8-piggyBac	0	1	0	1	89	54
9	0	0	0	0	8	7
10_ORF56	0	1	0	0	119	126
11-DNApol	0	1	0	0	72	96
12	0	0	0	0	21	21
13-OTU	0	1	0	0	26	19
14	0	0	0	0	5	6
15	0	0	0	0	15	11
16-ORF73_kinase	1	0	0	0	37	38
17	0	0	0	0	16	20
18-CD276	0	1	0	0	26	11
19-CD276	0	2	0	0	29	11
20	0	0	0	0	6	2
21-HERV-H_LTR-ass_pro_2	0	0	0	0	19	7
22	0	0	0	0	54	32
23-Mcl	0	0	0	0	10	3
24-CDK2	0	0	0	0	11	9
25-ZFP36	0	0	0	0	23	10
27	0	1	0	0	8	5
28-CAP_Gly	0	0	0	0	49	22
29	3	0	0	0	23	20
30	0	0	0	0	6	8
31-ORF44	0	0	0	0	28	14
32	0	0	0	0	34	33
33	0	0	0	0	11	12
34	0	0	0	0	19	11
35-ORF34	0	0	0	0	17	19
36	1	1	0	0	15	12
37	0	1	0	0	7	3
38-ORF37	0	1	0	0	32	33
39	0	0	0	0	14	5
40-MajorCapsid	0	1	0	0	43	62
41	0	0	0	0	18	29
42-Diguanylate_cyclase	3	0	0	0	11	8
43	0	0	0	0	33	25
44	0	1	0	0	62	27
45-MemGly	0	1	0	0	53	69
46	0	0	0	0	10	15
47-myopalladin	0	0	0	0	9	6
48-CapMat	0	0	0	0	33	30
49	0	0	0	0	12	12
50	0	0	0	0	14	10
51	0	0	0	0	11	6
52	0	0	0	0	1	4
53-Thiopurine_S_methylase	0	0	0	0	6	2
54-ZF_BED_domain	3	3	0	0	23	11
55-triplex	0	1	0	0	3	4
57	0	0	0	0	12	8
58-DNAhel	0	1	0	0	13	21
59	0	0	0	0	21	12
60-permease_YjgP/YjgQ	0	1	0	0	16	14
61-ZF_SCA	0	1	0	0	25	5
62-CXCR	0	0	0	0	18	6
63	0	0	0	0	23	14
64-TARBP	0	2	0	0	7	7
65	0	0	0	0	15	5
66	0	0	0	0	11	6

Supplementary Table 3 Continued

#GeneName	frameshift	stop_gained	inframe_del	inframe_ins	nonsynonymous	synonymous
67-ORF57R	0	0	0	0	14	13
68	0	0	0	0	4	2
69	0	0	0	0	31	23
70	2	3	0	0	73	26
71-methyltransferas	0	0	0	0	14	10
72-Primase	0	2	1	0	22	32
73	0	1	0	0	11	6
74-terminase	0	0	0	0	15	13
75	0	0	0	0	17	14
76	0	0	0	0	11	7
77	0	0	0	0	13	7
78-terminase	0	0	0	0	12	6
79-Cbp_p300	0	0	0	0	27	17
80-ORF70	0	0	0	0	23	28
81	0	0	0	0	4	3
82-ORF64	0	0	0	0	24	22
83	0	3	0	0	175	171
84	2	1	0	0	48	58
85	0	0	0	0	83	74
86	0	4	0	0	67	51
87	0	0	0	0	21	16
88	0	0	0	0	9	6
89-pim3	0	1	0	0	40	27
90-CLIC	0	0	0	0	6	4
**TOTAL**	**16**	**42**	**1**	**2**	**2251**	**1822**

Variant frequency	<0.1	0.1-0.25	0.25-0.5	0.5-0.9	0.9<

Supplementary Table 4 | Variants inside coding region of subtype 2 *Teratorn*.

#GeneName	frameshift	stop_gained	inframe_del	inframe_ins	nonsynonymous	synonymous
1L-HAFrepeat	0	0	0	0	1	0
2L-SIPA1	1	0	0	0	20	5
3L	1	1	0	0	13	3
4L	0	0	0	0	0	0
5L	1	0	0	0	0	2
6L-RdDp-jockey	3	1	0	0	13	9
7-RdDp-jockey	2	3	1	0	46	22
6R-RdDp-jockey-	1	0	0	0	3	3
5R	1	1	0	0	20	7
4R	1	3	0	0	16	10
3R	0	0	0	0	0	0
2R-SIPA1	0	0	0	0	8	0
1R-HAFrepeat	0	0	0	0	0	0
8	0	0	0	0	1	2
9-ORF60	0	0	0	0	6	9
10-ORF54	0	0	0	0	15	7
11-piggyBac	0	1	0	0	114	68
12-MyoD	0	0	0	0	41	17
13	4	1	0	0	8	4
14	0	0	0	0	5	2
15-DNAhel	0	1	0	0	3	7
16	0	0	0	0	1	1
17-triplex	0	0	0	0	3	6
18	3	0	0	0	2	2
19-ZF_BED	0	0	0	0	4	1
20	0	0	0	0	12	1
21	0	0	0	0	2	4
22	0	0	0	0	8	2
23	0	0	0	0	6	2
24	0	0	0	0	4	3
25-CapMat	0	0	1	0	7	6
26-myopalladin	0	0	0	0	5	2
27	0	0	0	0	3	2
28-MemGly	0	0	0	0	8	10
29	0	0	0	0	1	0
30	0	0	1	0	8	6
31	0	0	0	0	11	17
32	0	0	0	0	8	6
33	0	0	0	0	6	18
34-MajorCapsid	0	0	0	0	4	11
35	1	0	0	0	0	2
36	0	0	0	0	3	1
37-ORF37	1	0	0	0	4	10
38	1	0	0	0	10	5
39	0	0	0	0	2	5
40-ORF34	0	0	0	0	8	3
41	0	0	0	0	7	1
42	0	0	0	0	5	3
43-EH28	0	2	0	0	10	17
44-ORF44	0	0	0	0	9	10
45	2	1	0	0	24	11
46	0	0	0	0	25	26
47-CAP_Gly	0	1	0	0	82	33
48	4	0	0	1	4	2
49	0	0	0	0	18	9
50	0	0	0	0	3	3
51	2	0	1	0	52	61
52	0	0	0	0	35	16
53	0	0	0	0	2	0
54-ORF73_kinase	0	1	0	0	22	16
55	0	0	0	0	5	4
56-OTU	1	0	0	1	8	4
57	0	0	0	0	1	1

Supplementary Table 4 Continued

#GeneName	frameshift	stop_gained	inframe_del	inframe_ins	nonsynonymous	synonymous
58	0	1	0	0	2	4
59-DNApol	0	0	0	0	14	11
60-ORF56	1	0	0	0	54	59
61	0	0	0	0	5	3
62	0	0	0	0	3	1
63	0	0	0	0	0	1
64	0	0	0	0	2	2
65	0	0	0	0	7	9
66-TNFR	0	0	0	0	20	10
67-DNA_methyltransferase	0	0	0	0	4	3
68-primase	0	0	0	0	11	9
69	0	0	0	0	2	0
70-terminase1	0	0	0	0	1	3
71	0	0	0	0	3	7
72	0	0	0	0	4	2
73	0	0	0	0	4	2
74-terminase2	0	0	0	0	3	0
75-Cbp_p300	0	0	0	0	13	4
76-terminase3	0	0	0	0	15	26
77	3	0	0	0	5	2
78-ORF70	0	0	0	0	6	3
79	0	0	0	0	1	0
80-ORF64	0	0	0	0	11	4
81	0	2	0	0	62	33
82	0	0	0	0	8	3
83	1	0	0	0	4	3
84	0	0	0	1	26	15
85-Sorbitol_dehydrogenase	0	1	0	1	29	17
86	0	1	0	0	4	9
87-ORF78	0	0	0	0	4	4
88-pim	1	1	0	0	23	25
**TOTAL**	**36**	**23**	**4**	**4**	**1125**	**794**

Variant frequency	<0.25	0.25-0.75	0.75<

Supplementary Table 5 | Predicted ORFs inside *Teratorn* of *Oryzias dancena.*

Gene	properties or putative functions	Evalue	start	end	direction
ORF1			1	507	-
**ORF2**	**ORF60**	**1.00E-22**	**665**	**1948**	**+**
**ORF3**	**ORF54**	**9.00E-05**	**1939**	**3930**	**+**
**ORF4**	***piggyBac*-like transposase**	**0**	**4160**	**5842**	**+**
ORF6			7318	8655	+
**ORF7**	**DNA helicase (UL9 homolog)**	**2.00E-47**	**8708**	**10477**	**+**
ORF8			10558	10968	+
**ORF9**	**Capsid triplex protein subunit 2**	**6.00E-06**	**10994**	**11863**	**+**
ORF10	Zinc finger and BED domain-containing protein (L. crocea)	2.00E-20	12450	13172	-
ORF11			13645	14076	-
ORF12			14291	14818	-
ORF13			14834	15424	-
ORF14			15572	16645	-
**ORF15**	**Capsid maturation protease**	**2.00E-11**	**16983**	**19100**	**-**
ORF16	myopalladin, partial (O. latipes)	4.00E-09	19156	19737	-
ORF17			19935	20546	+
**ORF18**	**Membrane protein**	**3.00E-93**	**20679**	**24785**	**+**
ORF19			25822	27534	+
ORF20			27650	29329	+
ORF21			29939	30520	-
**ORF22**	**Major capsid protein**	**9.00E-22**	**30601**	**34053**	**-**
ORF23			34174	34608	-
**ORF24**	**ORF37**	**3.00E-08**	**34720**	**36969**	**+**
ORF25			36834	38210	-
ORF26			38273	39121	-
ORF27	ORF34	7.00E-19	39142	39891	-
ORF28			40409	41359	-
ORF29			41358	42083	+
ORF30			42258	45059	-
ORF31	ORF44	5.00E-18	45117	46388	+
ORF32			46433	47182	+
ORF33			47550	49193	+
ORF34			49225	51618	+
ORF35			51751	52281	+
ORF36	CDK2 (L. crocea)	1.00E-78	54322	55209	-
ORF37	Mcl1b	1.00E-10	55637	55945	-
ORF38			56874	60578	+
ORF39	CD276 antigen-like (H. burtoni)	1.00E-10	61870	62352	-
ORF40			66100	66561	-
ORF41	ubl carboxyl-terminal hydrolase (H. burtoni)	1.00E-28	68106	69020	-
ORF42	ser/thr protein kinase (. brichardi)	2.00E-27	69028	72474	-
ORF43			72516	73295	+
ORF44			73688	74290	-
ORF45	OTU-like cysteine protease (C. semilaevis)	5.00E-31	74701	76350	+
ORF46			76607	77740	-
**ORF47**	**DNA polymerase**	**2.00E-106**	**77912**	**82873**	**-**
ORF48			83088	83444	-
**ORF49**	**ORF56**	**3.00E-43**	**83537**	**87553**	**+**
ORF50	type2-angiotensin 2 receptor-like, CXCR (O. latipes)	2.00E-59	88092	88568	-
ORF51	type2-angiotensin 2 receptor-like, CXCR (O. latipes)	1.00E-36	88794	89168	-
ORF52			89802	90884	+
ORF53			91051	91515	+
ORF54			92819	93157	+
ORF55			94291	94644	-
ORF56			95202	95510	+
ORF57			96000	96689	-
ORF58	ORF57R (CyHV-3)	4.00E-11	97271	98464	-
ORF59			98607	99362	-
ORF60			99955	100764	-
ORF61			101366	102157	-
ORF62			102818	104377	-
ORF63	osteoprotegerin a precursor, TNFR-like (O. latipes)	1.00E-04	104374	108162	-
ORF64	DNA methyltransferase (Lymphocystis disease virus 1)		108365	109132	+
**ORF65**	**primase**	**7.00E-24**	**109786**	**112152**	**-**

Supplementary Table 5 Continued

Gene	properties or putative functions	Evalue	start	end	direction
**ORF66**	**DNA packaging terminase subunit 1**	**4.00E-38**	**112377**	**114260**	**-**
ORF67			114238	115113	+
ORF68			115247	116407	+
ORF69			116729	117256	+
**ORF70**	**DNA packaging terminase subunit 1**	**4.00E-11**	**117660**	**118493**	**-**
ORF71	Cbp-p300_interacting_transactivator1 (L. crocea)	3.00E-87	118689	119531	+
ORF72			120359	121570	+
ORF73			121951	122274	-
**ORF74**	**ORF64**	**2.00E-14**	**122255**	**123754**	**-**
ORF75			123751	129732	-
ORF76			129802	131241	+
ORF77			131312	135631	+
ORF78			136123	140157	-

Supplementary Table 6 | Predicted ORFs inside *Teratorn*-like element of yellow croaker (*Larimichthys crocea*).

Gene	Annotation	Evalue	start	end	direction
ORF1			121	609	+
ORF2			1734	2060	+
ORF3			2286	2888	+
ORF4	ORF72R (infectious spleen and kidney necrosis virus)	2.00E-36	3572	3961	-
ORF5	ORF72R (infectious spleen and kidney necrosis virus)	2.00E-38	3989	4390	-
ORF6			5426	5794	-
ORF7			6125	6427	-
ORF8	phosphatase_regulatory_subunit_15B (Xenopus tropicalis)	6.00E-07	6671	7006	-
ORF9			7332	8096	-
**ORF10**	**ORF60**	**4.00E-25**	**8603**	**9508**	**+**
**ORF11**	**ORF54**	**6.00E-04**	**9671**	**11671**	**+**
**ORF12**	**ORF64**	**6.00E-14**	**12416**	**14077**	**-**
ORF13			14080	14748	-
ORF14			14790	20546	-
ORF15			20754	22091	+
ORF16			22260	26363	-
ORF17			26833	31038	-
ORF18			31280	32782	-
ORF19			32781	34226	+
ORF20			34990	37461	+
ORF21			38048	38437	+
ORF22			38566	39261	+
ORF23			39454	39861	+
ORF24			40287	40850	-
**ORF25**	**Major capsid protein**	**1.00E-49**	**40967**	**44395**	**-**
ORF26			44558	44947	-
ORF27			45119	46072	+
**ORF28**	**ORF37**	**5.00E-10**	**46072**	**47391**	**+**
ORF29			47950	49269	-
ORF30			49303	50166	-
**ORF31**	**ORF34**	**9.00E-43**	**50258**	**51724**	**-**
ORF32			51567	52565	-
ORF33			52864	53310	+
ORF34			53622	55967	-
**ORF35**	**ORF44**	**2.00E-16**	**56181**	**57728**	**+**
ORF36			57837	58565	+
ORF37			58757	60127	+
ORF38	GRIP_and_coiled_coil (N. brichardi)	4.00E-18	60124	63150	+
ORF39			64474	65001	-
ORF40			65112	65762	-
ORF41	Thioredoxin domain-containing protein 16 (L. crocea)	7.00E-40	66275	66724	+
ORF42	E3_ubiquitin_ligase_makorin_like (S. partitus)	1.00E-24	67498	67824	-
ORF43			68413	69207	-
ORF44			69284	69937	+
ORF45	ORF74_protein_kinase (IcHV-1)	3.00E-08	69822	72800	+
ORF46			74045	77260	-
ORF47			77354	77656	-
ORF48	ribonuclease3 (Firmicutes bacterium)	5.00E-06	79033	79557	-
ORF49			80066	80425	+
ORF50			80467	80859	+
ORF51			82345	83298	-
ORF52	CDK2-like (O. latipes)	3.00E-79	83720	84652	+
ORF53			85901	86272	+
ORF54			86450	87295	+
**ORF55**	**DNA helicase (UL9 homolog)**	**1.00E-32**	**87369**	**89156**	**+**
ORF56			89556	89942	+
**ORF57**	**Membrane protein**	**9.00E-89**	**90410**	**94528**	**-**
ORF58			94667	95584	-
ORF59			95527	96105	+
**ORF60**	**Capsid maturation protease**	**4.00E-12**	**96219**	**98195**	**+**
ORF61			98503	99570	+
ORF62			99630	100226	+
ORF63			101040	101606	+
**ORF64**	**Capsid triplex protein subunit 2**	**1.00E-05**	**102451**	**103074**	**-**

Supplementary Table 6 Continued

Gene	properties or putative functions	Evalue	start	end	direction
ORF65			103170	103604	-
ORF66			103966	108681	-
ORF67			109352	109795	+
ORF68	ORF74 (Infectious spleen and kidney necrosis virus)	8.00E-15	110131	111033	+
ORF69			112595	113608	-
ORF70	ORF35_AngHV	4.00E-13	113973	114986	-
ORF71	extensin1-like (C. carolinensis)	7.00E-05	115106	116218	-
ORF72			116853	118598	-
ORF73			118579	120915	-
ORF74	TNFR_superfamily_6B-like (E. lucius)	0.032	121306	122730	-
ORF75	Cytosine_methyltransferase (Turbot reddish body iridovirus)	1.00E-07	122796	123674	+
**ORF76**	**Primase**	**3.00E-18**	**123869**	**126163**	**-**
**ORF77**	**DNA packaging terminase subunit 1**	**3.00E-39**	**126482**	**127624**	**-**
ORF78			127623	128456	+
ORF79	FK506_binding_protein (O. niloticus)	7.00E-05	129332	129679	+
ORF80	type4_pilus_biogenesis_protein		129810	130286	+
ORF81	Pyrin_domain_containing_protein1 (O. hannah)	0.017	131318	131866	-
**ORF82**	**DNA packaging terminase subunit 1**	**5.00E-11**	**132348**	**133175**	**-**
ORF83	Cbp-p300_interacting_transactivator1 (L. crocea)	0	133266	134069	+
**ORF84**	**DNA packaging terminase subunit 1**	**7.00E-06**	**134031**	**134330**	**-**
ORF85			134929	136161	+
**ORF86**	**ORF56**	**1.00E-42**	**136608**	**140609**	**-**
ORF87	Cas5-like (S. aarchaeon)	0.059	141087	141386	-
**ORF88**	**DNA polymerase**	**5.00E-99**	**141593**	**146752**	**+**
ORF89			146806	147969	+
ORF90	OTU-like cysteine protease (O. niloticus)	4.00E-48	149441	151249	-
ORF91			151552	152010	+
**ORF92**	***piggyBac*-like transposase**	**5.00E-106**	**152426**	**156105**	**-**
ORF93			156110	156412	-

Supplementary Table 7 | Predicted ORFs inside *Teratorn*-like element of nile tilapia (*Oreochromis niloticus*).

Gene	Annotation	Evalue	start	end	direction
**ORF1**	***pgigyBac***	**0**	**394**	**3354**	**-**
ORF2			4171	4965	-
ORF3			5158	5625	-
ORF4			6343	7086	+
ORF5	MHC class I antigen (O. niloticus)	0	7730	8842	+
ORF6	ORF147B (CyHV-1)	8.00E-13	9439	10497	-
ORF7	ORF147A, TNFR-like (CyHV-1)	2.00E-09	10880	12295	+
ORF8	ORF147A, TNFR-like (CyHV-1)	5.00E-48	12283	12996	+
ORF9	butyrophilin-like (O. niloticus)	3.00E-29	13639	14250	-
ORF10			14371	15051	+
ORF11	MHC class I antigen, KD alpha chain-like (N. brichardi)	6.00E-04	15348	15686	-
ORF12			15806	16108	-
ORF13	LPXTG-motif cell wall anchor domain (L. gasseri)	2.00E-12	16509	16988	-
ORF14	MHC class I antigen, KD alpha chain-like (N. brichardi)	2.00E-55	17056	17766	+
ORF15			18270	18584	+
ORF16	MHC class I antigen, KD alpha chain-like (N. brichardi)	7.00E-39	18672	18998	+
ORF17	coxsackievirus and adenovirus receptor homolog (N. brichardi)	1.00E-10	19033	19533	-
ORF18			20201	20563	+
ORF19	MHC class I antigen, KD alpha chain-like (N. brichardi)	1.00E-45	20596	20946	+
ORF20	coxsackievirus and adenovirus receptor homolog (N. brichardi)	3.00E-34	21105	21671	-
ORF21			22247	22759	+
ORF22			22966	23529	+
ORF23	E3 ubiquitin ligase TRIM31	2.00E-04	23562	23879	+
ORF24			24004	24711	-
ORF25	GRIP and coiled-coil domain (N. brichardi)	0	24720	26150	-
ORF26			26215	27951	-
ORF27			28174	28971	-
**ORF28**	**ORF44**	**6.00E-17**	**28987**	**30375**	**-**
ORF29			30229	32337	+
ORF30			32275	33171	-
ORF31			33418	33771	+
ORF32	interleukin-2 receptor subunit beta (M. zebra)	4.00E-06	33775	34551	+
**ORF33**	**DNA polymerase (IcHV-1)**	**5.00E-38**	**34877**	**36763**	**+**
**ORF34**	**DNA polymerase**	**8.00E-13**	**36751**	**37323**	**+**
ORF35			37423	37866	+
**ORF36**	**DNA polymerase**	**3.00E-05**	**37944**	**38276**	**+**
**ORF37**	**DNA polymerase**	**2.00E-04**	**38303**	**39007**	**+**
ORF38			39037	39765	+
ORF39			39936	41135	+
ORF40	OTU-like cysteine protease (O. niloticus)	4.00E-27	41325	42992	-
ORF41			43506	43901	-
**ORF42**	**Capsid maturation protease**	**2.00E-05**	**44502**	**45152**	**-**
ORF43			45193	45750	-
ORF44			45939	46529	+
**ORF45**	**Membrane glycoprotein**	**2.00E-98**	**46598**	**50215**	**+**
ORF46			50161	50643	+
ORF47			51254	52024	+
ORF48			52318	52896	-
**ORF49**	**Major capsid protein**	**5.00E-48**	**52943**	**56320**	**-**
ORF50			56359	56769	-
**ORF51**	**ORF37**	**1.00E-08**	**56896**	**59178**	**+**
ORF52			59209	60093	-
ORF53			60096	60557	-
ORF54			60560	60910	-
**ORF55**	**ORF34**	**2.00E-42**	**60991**	**62469**	**-**
ORF56			62885	63874	+
ORF57			64143	65333	+
ORF58	coxsackievirus and adenovirus receptor homolog (N. brichardi)	1.00E-04	66154	66639	+
ORF59			67538	68032	-
ORF60			68174	68974	-
ORF61	Fc receptor-like protein 3 (M. zebra)	1.00E-57	69978	70829	-
ORF62			70986	71306	-
ORF63			72358	73236	-
ORF64			73445	74011	-

Supplementary Table 7 Continued

Gene	properties or putative functions	Evalue	start	end	direction
ORF65	FK506-binding protein 4-like (O. niloticus)	1.00E-138	74023	74718	-
ORF66			74947	75942	+
ORF67			77135	78280	-
ORF68			78972	79487	+
ORF69			79677	80429	-
ORF70	interleukin-2 receptor subunit beta (M. zebra)	1.00E-05	81201	81917	+
ORF71			82245	83378	+
ORF72	CDK-like (O. niloticus)	9.00E-176	83401	84333	+
ORF73			84934	85977	-
ORF74			86167	86913	+
ORF75			86977	87396	+
ORF76			87877	88728	-
ORF77			88839	89831	-
**ORF78**	**DNA packaging terminase subunit 1**	**1.00E-39**	**89896**	**90798**	**+**
**ORF79**	**Primase**	**6.00E-30**	**91030**	**93069**	**+**
ORF80	myelin-oligodendrocyte glycoprotein	3.00E-73	93858	94346	-
ORF81			94675	95013	-
ORF82			95304	96185	-
ORF83	ORF85_LPXTG_motif	7.00E-15	96396	97112	-
ORF84			97455	98198	+
ORF85			98372	99730	+
ORF86			99846	101507	+
ORF87	apoptosis-associated_speck-like protein (L. crocea)	0.027	102202	102816	+
ORF88			102974	103315	+
ORF89	ribonuclease 3 (C. testudinoris)	0.033	104042	104566	-
ORF90			105407	108829	+
ORF91			109514	110122	+
ORF92	ubl carboxyl terminal hydrolase 1 (H. burtoni)	3.00E-178	110818	111768	-
ORF93	ser/thr protein kinase	2.00E-10	112426	114444	-
ORF94			114465	115418	-
ORF95			115567	116694	+
ORF96	E3 ubiquitin ligase makorin-like(T. chinensis)	5.00E-31	117022	117435	+
ORF97			117888	118505	-
ORF98	coiled-coil domain-containing protein 86 (P. anubis)	0.011	118484	118906	-
ORF99	cyclin-related protein (P. yoelii)		119002	119727	-
ORF100			119846	120220	+
**ORF101**	**DNA packaging terminase subunit 1**	**8.00E-04**	**120459**	**121193**	**-**
ORF102	Cbp/p300-interacting transactivator 1 (L. crocea)	9.00E-83	121354	122235	+
ORF103			122802	123578	+
ORF104			123575	124099	+
ORF105			124389	124688	-
**ORF106**	**ORF64**	**1.00E-07**	**124699**	**126279**	**-**
ORF107			126347	127150	-
ORF108			126985	132057	-
ORF109			132016	133371	+
ORF110			133496	136498	+
ORF111			136527	137174	+
ORF112			137684	138280	-
ORF113			138282	142223	-
ORF114			142426	143271	-
ORF115			143333	143803	-
ORF116	ORF78 (IcHV-1)	0.42	143784	145220	+
ORF117			146929	147312	+
ORF118	ORF35 (AngHV-1)	0.4	151398	151742	-
ORF119			151861	152418	-
ORF120			154311	154637	-
ORF121			154638	155144	-
ORF122			155407	155790	-
ORF123			156788	157330	-
ORF124			157263	158930	-
ORF125	DNA methyltransferase (Euryarchaeota archaeon)	3.00E-15	159033	159887	+
ORF126			160058	160564	-
**ORF127**	**ORF56**	**2.00E-34**	**160751**	**163183**	**+**
**ORF128**	**ORF56**	**7.00E-06**	**163153**	**164724**	**+**
ORF129	CXCR-like (O.latipes)	2.00E-12	165041	165454	-
ORF130	CXCR-like (P. formosa)	2.00E-10	165707	166111	-
ORF131	MHC class I antigen, KD alpha chain-like (N. brichardi)	0.002	166848	167330	+

Supplementary Table 7 Continued

Gene	properties or putative functions	Evalue	start	end	direction
**ORF132**	**ORF54**	**0.084**	**167679**	**168074**	**-**
ORF133			168356	169252	-
**ORF134**	**ORF60**	**9.00E-18**	**169750**	**170202**	**-**
ORF135			170682	171425	+
ORF136			172632	173885	+
**ORF137**	**DNA helicase (UL9 homolog)**	**5.00E-47**	**173899**	**175683**	**+**
ORF138			175738	176136	+
**ORF139**	**Capsid triplex protein subunit 2**	**0.007**	**176409**	**177035**	**+**
ORF140			177978	178496	-
ORF141			178534	179109	-
ORF142			179189	180238	-
ORF143	MHC class I antigen, alpha (O. latipes)	3.00E-31	180687	181424	-
ORF144			181948	182808	+
ORF145			183948	184325	-
ORF146			187046	188065	-
ORF147			188686	189111	+
ORF148			190893	191699	+
ORF149	probable Bax inhibitor 1-like (O. niloticus)	2.00E-179	191854	192627	-
**ORF150**	***piggyBac***	**6.00E-135**	**193330**	**195923**	**-**

Supplementary Table 8 | List of medaka strains used in this analysis.

Species	Collection site
Hd-rR*, O. latipes*	Aichi, Japan
HNI*, O. latipes*	Niigata, Japan
O. curvinotus	Sam A tsuen, Plover Cove Country Park, Hong Kong
O. luzonensis	Solsona, Ilocos Norte Province, Philippines
O. mekongensis	Udon Thani, Thailand
O. dancena	Chidambaram, Tamil Nadu, India
O. javanicus	Penang, Malaysia
O. celebensis	Malino river, Sulawesi, Indonesia
O. sarasinorum	Lake Lindu, Sulawesi, Indonesia
O. nigrimas	Tentena, Lake Poso, Sulawesi, Indonesia
O. matanensis	Soroako, Lake Matano, Sulawesi, Indonesia
O. marmoratus	Timampuu, Lake Towuti, Sulawesi, Indonesia
O. profundicola	Timampu, Lake Towuti, Sulawesi, Indonesia

Supplementary Table 9 | Primer sequences used in this analysis.

Experiment	Name	Primer sequence
BAC Screening	Tera_LeftR1	GTAAAACAGCGGAGGGAATGGGTTTCGTGC
	Tera_LeftR2	AGACCCCAAAGCACATGCCAACCTAAG
	Tera_RightF	GGTGTACCTGTCTGGGGTCAATACCAAAGG
	13I17_LeftF	AAGGCTGTGTCCCTCTGCAATGAGACTGTTA
	13I17_RightR	GACAGCTACAAGTCTTGGTATGAGAGAGTC
	14A10_LeftF	CTCTGGATGGAAAAGACTTCTAACCAAGTGG
	14A10_RightR	GTCCAGAAAAGCTGTTTTACACGGAGTCCTA
	6L21_LeftF	ACAGTGCTTCATAGAGAAACGCTTCCAC
	6L21_RightR	CAGTGTTTGGGATCTTTGAAAAGGTCAG
	11H24_LeftF	AGAATGAGCTCCAACAATCCCTTCATGC
	11H24_RightF	AAACCAGTGAGGCATTACCAGCTTTTGC
	11H24_RightR	TGACGAAGAAATACGGTATCCAGCTGTC
	73I9_LeftF	GGGAATGGTGTGAACAGTTTGGAGTTTC
	73I9_RightF	TCCACATAGTGCCATCTAGGATTTCAGG
	73I9_RightR	GGAGCGGAGTAAGCAGTTTTACATAACCC
	85H23_LeftF	GTTGTGTCACTACGCATTCTTTCTCACC
	85H23_RightR	GTCTTTCAAAATCCACACAGGCCAGGTC
Indicator plasmid	AcGFP_in_fusion_F	GGACTCAGATCTCGAGCATTGATGAATGAGACGGCTTTG
	AcGFP_in_fusion_R	GTCGACTGCAGAATTCTGTACTCATTGAACTTTGGATGGTC
	Sub1_LeftTIR_F	CATTGATGAATGAGACGGCTTTGATTTGGA
	Sub1_LeftTIR_R	TTATCGATCCCTGGTGGTCGTGCGCTGTAAG
	Sub1_puroGFP_F	CACCAGGGATCGATAACCGTATTACCGCCATGC
	Sub1_puroGFP_R	CGCTTTACCCGCCTTTGAGTGAGCTGATACCGC
	Sub1_RightTIR_F	AAAGGCGGGTAAAGCGAGACCCCAGGTGGTG
	Sub1_RightTIR_R	TGTACTCATTGAACTTTGGATGGTCACACA
	Sub1_internal_TIR_F	gaacatcaATCGATCTGGTGGTCGTGCGCTGTAAG
	Sub1_internal_TIR_R	gttacgttATCGATCGGATTACTGTGTCAAAGTGC
	Sub2_LeftTIR_F	CAGTAAGGCCAGTAGATGTAGCGGATGTG
	Sub2_LeftTIR_R	GTTATCGATTCGTAGAAGTAAGGTTCGGGTCGCGTG
	Sub2_puroGFP_F	CTTCTACGAATCGATAACCGTATTACCGCCATGC
	Sub2_puroGFP_R	ACCTGATGCCGCCTTTGAGTGAGCTGATACCGC
	Sub2_RightTIR_F	ACCTGATGCCGCCTTTGAGTGAGCTGATACCGC
	Sub2_RightTIR_R	ACCTGATGCCGCCTTTGAGTGAGCTGATACCGC
	Sub2_internal_TIR_F	GAACATCAATCGATCTCATAGAAGTAAGAGTGGGGTC
	Sub2_internal_TIR_R	GTTACGTTATCGATTAGATCACGTGAGGTGAATGAC
Helper plasmid	Sub1_TPase_CDS_Kozak_F	GGAATTCCGCCACCATGAACAAAGGCCGCAAAAGAAC
	Sub1_TPase_CDS_R	GTCGACTCACTGGGACGCGTCTGTG
	Sub2_Tpase_CDS_Kozak_F	GAATTCCGCCACCATGGGTCCCAAAAGACCTCAAAG
	Sub2_TPase_CDS_R	GGTCGACCTAGTCTGTGTCACTGTTGGAATCG
(EPTS)LM-PCR	LMPCR-adaptor-long-oligo	GACCCGGGAGATCTGAATTCAGTGGCACAGCAGTTAGG
	LMPCR-adaptor-short-oligo	(p)CCTAACTGCTGTGCCACTGAATTCAGATCTCCC
	Sub1-LMPCR-LeftTIR-biotin-oligo-F	(bio-) TGTAAAACAGCGGAGGGAATGGAGAG
	Sub2-LMPCR-LeftTIR-biotin-oligo-F	(bio-) TGACAATCGAGTGAACATTCTGACAG
	Sub1-LMPCR-LeftTIR-primer	AACCTAAGCGAGCGGAAG
	Sub2-LMPCR-LeftTIR-primer	ACCCTTCATTACAGCGTAGG
	LMPCR-common-primer	GACCCGGGAGATCTGAATTC
RT-PCR	piggyBac_F	TCAGAGAGGTGTGGGAAGAGTG
	piggyBac_R	GTCATTCTCCTGCAGCTGTACG
	DNA_polymerase_F	CACAGGTGCAGGATCACGCTCAACATAG
	DNA_polymerase_R	GGAGGATCAGCGTCGCATACTCAAAGC

Experiment	Name	Primer sequence
RT-PCR	DNA_helicase_F	CACCGTGTTGAACTGGAGGTAGGAGAGTG
	DNA_helicase_R	TTTGTCGAGCGCTACAGGAGATGCTACC
	Terminase_F	GTTGCTGCTGGACAGAAAGTGAGCGAAG
	Terminase_R	GATGGAGCTCTTGAGAGGAGTCGTGCTG
	Major_capsid_F	TTCAAGCAGCAGAAGGACACGAGC
	Major_capsid_R	TCAGCACAGAGTCCAGCTTCTCC
	Capsid_triplex_F	ACCGCTACGTTCCCACTCTACAC
	Capsid_triplex_R	GAATCTGTCGTTCACCAAAGACG
	Membrane_glycoprotein_F	GCGTCCCTGAGGAGTCCAAAGTTCTTG
	Membrane_glycoprotein_R	AGAGACTACGGCAAGCTGTGCGATTCAG
	ZFP36_F	TCTGAACACCCAACAGCTCTCA
	ZFP36_R	GGAAACGTCCATCTGTCGGTAG
	CXCR_F	GTTCAGTGTGGTACCCATGCTTC
	CXCR_R	CGACTTTGAAGGTTTCGTGATTG
	DNA_methyltransferase_F	CTCGTCCTCTTTCGGTACTTGG
	DNA_methyltransferase_R	GAGACACGGGGCTGATATAGTGA
	CDK_F	AGTTGTCTACAGGGTGCGAGATG
	CDK_R	CTCCCAAACTCCACATGTCTACG
	pim_F	GCAAAGTGTTTCTGGGAAAGAGG
	pim_R	TGTCGTTTCCAAATGGTATGTCC
	ZnSCAN_F	TGGAGCGATGATGAGGTTAAATG
	ZnSCAN_R	ATAAATTCCGTTTGCCCTTACCTG
	bactin_F	GATGAAGCCCAGAGCAAGAG
	bactin_R	AGGAAGGAAGGCTGGAAGAG
qRT-PCR	piggyBac_qPCR_F	GGTTCCTTTCAAAGGACGTTG
	piggyBac_qPCR_R	CCTCCAAGCGTAGCTCGTC
	DNA_polymerase_qPCR_F	CGATTTCGCCAGCATGTACC
	DNA_polymerase_qPCR_R	ACTTGTACACGACCCATCCG
	DNA_helicase_qPCR_F	CAGCAGGATCTTCGCTCGG
	DNA_helicase_qPCR_R	GTCATGAGCCCGTACTCGTC
	Terminase_qPCR_F	CTTGAGAGGAGTCGTGCTGG
	Terminase_qPCR_R	CGGGTCCTGGTAATCGTAGC
	Major_capsid_qPCR_F	GGTTCACGAGGCGTTACAAG
	Major_capsid_qPCR_R	GGTTCACGAGGCGTTACAAG
	Capsid_triplex_qPCR_F	CGCTACGTTCCCACTCTACA
	Capsid_triplex_qPCR_R	TTCAGGAACGGTCTGTCCAC
	Membrane_glycoprotein_qPCR_F	TCTGGCATTGCACGTGTCTC
	Membrane_glycoprotein_qPCR_R	GTTCTTGTTGGCGATCTCGG
	ZFP36_qPCR_F	GATATGTTCACCAGAGCTGACAC
	ZFP36_qPCR_R	GGTAGCCGTCCTTGTTCAGA
	CXCR_qPCR_F	GGTGATAACTTTCTGCCTGC
	CXCR_qPCR_R	GATATGTTCACCAGAGCTGACAC
	DNA_methyltransferase_qPCR_F	CTCCGTGCGAAAACTACAGC
	DNA_methyltransferase_qPCR_R	TGCGAGGGTTTTGTCCATGA
	CDK_qPCR_F	CAAAGCTATTCACCGCCAGC
	CDK_qPCR_R	AGCACGTTACTCGGAACCAG
	pim_qPCR_F	CTCCTACTGGGCGAGCTTAG
	pim_qPCR_R	TCAGCATCGGTCTCTCTTCG
	ZnSCAN_qPCR_F	GGTAGCCGTCCTTGTTCAGA
	ZnSCAN_qPCR_R	GCCACGAGCGCTTAAAACC
	bactin_qPCR_F	TGCCGCACTGGTTGTTGACAACG
	bactin_qPCR_R	CCAGGCACCAGGGTGTCATGG

Experiment	Name	Primer sequence
Antibodies	Major_capsid_GST_F	AAGAATTCGTGCGAGGGGGAGACGTG
	Major_capsid_GST_R	AAGTCGACCTACTGCATGATGTCCGTGGC
	Triplex_GST_F	AAGAATTCGACAGCAACCGCTACGTTC
	Triplex_GST_R	AAGTCGACCTACATGCTTGAGCGCCTGC
	Membrane_glycoprotein_GST_F	AAGAATTCAAGGAGCCCCACAGGGAG
	Membrane_glycoprotein_GST_R	AAGTCGACCTACGCCGGTTTCACGATCAC
Medaka related species	DNA_polymerase_deg_F1	GTAARCTNYDSAAGCTRRCSGRCGT
	DNA_polymerase_deg_F2	GCSGTGCTBSTSTACAACTTGGTBG
	DNA_polymerase_deg_R1	CCAKGACTTTRCACTCYMCCTCCTG
	DNA_polymerase_deg_R2	CKGTTGAGRTARTRCTCNACMGAGG
	Major_capsid_deg_F1	TCACSKTNGACGAMRTSGACTAYTG
	Major_capsid_deg_F2	CVGGCWTGTCSTAYCTRMTGTTCAG
	Major_capsid_deg_F3	GGCWTGTCSTAYCTRHTGTTCAG
	Major_capsid_deg_R1	CTGAACAKYAGRTASGACAWGCCBG
	Major_capsid_deg_R2	GTNGTGAARATGTAVGGYCTGCGGT
	Major_capsid_deg_R3	TAAGTACCRCAGAARGCMACYCCNA
	Major_capsid_deg_R4	AGTACCDCAGAARGCMACYCCNA
	Membrane_glycoprotein_deg_F1	GCNATTTGCCTSAGACAGTTYTCHA
	Membrane_glycoprotein_deg_F2	CRTCATYTGATGSGTCACGCTYTGC
	Membrane_glycoprotein_deg_F3	GCGTCYCTGAGGAGTCCRAAGTTYTTG
	Membrane_glycoprotein_deg_R1	GTGAACGACRGNTACRYGNTGGAYA
	Membrane_glycoprotein_deg_R2	CCCACSASNGAYMSCARTCACTAYA
	Membrane_glycoprotein_deg_R3	AGAGACTACGGCAAGCTGTGCGATTCRG
	DNA_helicase_deg_F1	AGTTCATCTCCACBCKTTTRCGRTA
	DNA_helicase_deg_F2	TARAAMNCMACMCGWCCSACNKWST
	DNA_helicase_deg_F3	RGTTCATCTCCWCBCKTTYRSGRKW
	DNA_helicase_deg_R1	GGCACATGGAYYMSAACGGNATACA
	DNA_helicase_deg_R2	TSTTTGTCAACAGRMGRCAGTTCSA
	DNA_helicase_deg_R3	TSTTTRTCAACAGRMGRCARTTCSA
	Terminase_deg_F1	ATCTCRTCTCTKGTGTGRCAHAGRA
	Terminase_deg_F2	TACATGWRCACGKCCATRKWGGAGC
	Terminase_deg_F3	AWCTCDTCYCTKGTGTGRCAHAAVA
	Terminase_deg_F4	TACATGHVCACRKYCATRKNSGANC
	Terminase_deg_F5	TYTCRTCTCTKGTGTGRCANAGVA
	Terminase_deg_R1	ATCTCRTCTCTKGTGTGRCAHAGRA
	Terminase_deg_R2	TACATGWRCACGKCCATRKWGGAGC
	Terminase_deg_R3	ARRTACATGYTVGGRAARCACGTNR
	Terminase_deg_R4	RGTACATGCTRGGRAARCACGTSR
	piggyBac_F	TCAGAGAGGTGTGGGAAGAGTG
	piggyBac_R	GTCATTCTCCTGCAGCTGTACG
	Major_capsid_sub2_F	CCGGCTTGTCCTATCTGTTGTTCAG
	Major_capsid_sub2_R	TAAGTACCACAGAAAGCCACCCCGA
	Membrane_glycoprotein_sub2_F	AGAGACTACGGCAAGCTGTGCGATTCAG
	Membrane_glycoprotein_sub2_R	GCGTCTCTGAGGAGTCCGAAGTTTTTG
